# Supplementary material for: Mass spectrometry imaging of SOD1 protein-metal complexes in SOD1G93A transgenic mice implicates demetalation with pathology
Source: Nat Commun. 2024 Aug 8;15:6518. doi: 10.1038/s41467-024-50514-7 (PMC11310518; doi:10.1038/s41467-024-50514-7)
Supplement: Supplementary file 1 — Supplementary information [file 41467_2024_50514_MOESM1_ESM.pdf]

Supplementary Information for "Mass spectrometry imaging of SOD1 protein-metal complexes in SOD1^{G93A} transgenic mice implicates demetalation with pathology"

Oliver J. Hale[1], Tyler R. Wells[2], Richard J. Mead[2] and Helen J. Cooper[1]

Affiliations:
1: School of Biosciences, University of Birmingham, Birmingham, B15 2TT, UK.
2: Sheffield Institute for Translational Neuroscience, University of Sheffield, S10 2HQ

Supplementary Figures

Figure S1: amino acid sequences for mSOD^{wt}, hSOD1^{wt} and hSOD1^{G93A} S3
Figure S2: hSOD1^{G93A} protein complexes detected by NAMS - summary S5
Figure S3: nano-DESI-SIM mass spectrum from hSOD1^{G93A} transgenic mouse brain. S6
Figure S4: nano-DESI-SIM mass spectrum from hSOD1^{wt} transgenic mouse brain. S7
Figure S5: hSOD1^{wt} nano-DESI-HCD MS^2 ....S9
Figure S6: hSOD1^{G93A} (holo) nano-DESI-HCD MS^2...S10
Figure S7: hSOD1^{G93A} (metal-deficient) nano-DESI-HCD MS^2 ....S11
Figure S8: hSOD1^{G93A} nano-DESI-HCD MS^3 ....S12
Figure S9: hSOD1^{G93A} (holo) nano-DESI-EThcD MS^2...S13
Figure S10: hSOD1^{G93A} (metal-deficient) disulfide bond (nano-DESI-HCD MS^2) S14
Figure S11: hSOD1^{G93A} SIM vs HCD neutral loss ions..S16
Figure S12: hSOD1^{G93A} (6$^+$ monomer) nano-DESI-PTCR MS^2 ....S17
Figure S13: Denatured hSOD1^{G93A} LESA-HCD MS^2..S18
Figure S14: SIM vs calculated spectrum for xNa$^+$ adduct formation S20
Figure S15: Ion images for protein charge states for hSOD1^{G93A} spinal cord S21
Figure S16: Ion images for protein charge states for hSOD1^{wt} spinal cord. S22
Figure S17: Ion images for monomers in hSOD1^{G93A} and hSOD1^{wt} spinal cord....................S22
Figure S18: FFPE spinal cord section, Nissl stain. S23
Figure S19: Ion images for hSOD1^{wt} dimers in charge states 11+ - 9+, whole brain S24
Figure S20: Ion images for protein charge states in hSOD1^{wt} brain, high-resolution S25
Figure S21: Ion images for hSOD1^{G93A} dimers in charge states 11+ - 9+, whole brain S26
Figure S22: Ion images for protein charge states in hSOD1^{G93A} brain, high-resolution S27
Figure S23: hSOD1^{wt} brain biological replicates (dimers)...S28
Figure S24: hSOD1^{G93A} brain biological replicates (dimers)..S28
Figure S25: whole brain biological replicates (hSOD1^{wt}, hSOD1^{G93A} monmers)...................S29
Figure S26: FFPE brainstem, Nissl stain S30
Figure S27: 2GS-hSOD1^{G93A} ion images, replicates..S30
Figure S28: 1GS-hSOD1^{G93A} ion images (dimers), replicates. S31
Figure S29: High-resolution NAMS imaging of hSOD1^{G93A} in cerebellum – charge states. ...S32
Figure S30: non-SOD1 proteins in brain sections, replicates...S33
Figure S31: high-resolution MS of hSOD1^{G93A} for dimer/monomer overlap estimation. S33

Supplementary Tables

Table S1: Animals included in the study. S3
Table S2: Calculated and measured molecular weights (MW) for hSOD1G93A and hSOD1wt dimer complexes...S8
Table S3: sequence ions for HCD MS^3 analysis of hSOD1^{G93A}..S12
Table S4: sequence ions for EThcD MS^2 analysis of hSOD^{G93A}...S13
Table S5: sequence ions for hSOD1^{G93A} with an intact intramolecular disulfide bond from metal-deficient dimers...S15
Table S6: HCD product ions from m/z 1078^{15+} S19
Table S7: Parameters for deconvolution with UniDec. S33

Supplementary Note 1: Identification and characterization of protein complexes...............S4

Supplementary Tables and Figures

```
                                   m/z 1158.6
sp|P08228|SODC_MOUSE               AMKAVCVLKGDGPVQGTIHFEQKASGEPVVLSGQITGLTEGQHGFHVHQYGDNTQGCTSA 60
sp|P00441|SODC_HUMAN               ATKAVCVLKGDGPVQGIINFEQKESNGPVKVWGSIKGLTEGLHGFHVHEFGDNTAGCTSA 60
sp|P00441|SODC_HUMAN_G93A          ATKAVCVLKGDGPVQGIINFEQKESNGPVKVWGSIKGLTEGLHGFHVHEFGDNTAGCTSA 60
                                   * ************** *:**** *. ** : *.*.***** ******::**** *****
                                   m/z 1128.6

sp|P08228|SODC_MOUSE               GPHFNPHSKKHGGPADEERHVGDLGNVTAGKDGVANVSIEDRVISLSGEHSIIGRTMVVH 120
sp|P00441|SODC_HUMAN               GPHFNPLSRKHGGPKDEERHVGDLGNVTADKDGVADVSIEDSVISLSGDHCIIGRTLVVH 120
sp|P00441|SODC_HUMAN_G93A          GPHFNPLSRKHGGPKDEERHVGDLGNVTADKDAVADVSIEDSVISLSGDHCIIGRTLVVH 120
                                   ****** *:***** **************.**.**:***** ******:*.*****:***

sp|P08228|SODC_MOUSE               EKQDDLGKGGNEESTKTGNAGSRLACGVIGIAQ 153
sp|P00441|SODC_HUMAN               EKADDLGKGGNEESTKTGNAGSRLACGVIGIAQ 153
sp|P00441|SODC_HUMAN_G93A          EKADDLGKGGNEESTKTGNAGSRLACGVIGIAQ 153
                                   ** ******************************
```

Figure S1: aligned amino acid sequences for wild-type mouse (mSOD1^{wt}), wild-type human (hSOD1^{wt}) and the human variant G93A (hSOD1^{G93A}) SOD1. The second amino acid differs between mouse and human SOD1 (cyan highlight). The b_{11}^{+} fragment ion (m/z 1158.6, mouse; m/z 1128.6, human) is favoured under collisional activation and thus is useful for confirming the presence of human SOD1. The G93A mutation is highlighted in yellow.

Table S1: Animals included in the study.

ID Code[a]	Age (days)	Sex	Description
**G93A-B1**	120	M	Transgenic (hSOD1^{G93A})
**G93A-B2**	124	F	Transgenic (hSOD1^{G93A})
**G93A-B3**	124	F	Transgenic (hSOD1^{G93A})
**hWT-B1**	180	M	Transgenic (hSOD1^{wt})
**hWT-B2**	180	M	Transgenic (hSOD1^{wt})
**hWT-B3**	150	M	Transgenic (hSOD1^{wt})
**G93A-SC1**	120	F	Transgenic (hSOD1^{G93A})
**G93A-SC2**	120	F	Transgenic (hSOD1^{G93A})
**hWT-SC1**	150	M	Transgenic (hSOD1^{wt})
**hWT-SC2**	150	M	Transgenic (hSOD1^{wt})
**mSODwt FFPE (n=5)**	120 ± 3	F	[b]Wild-type (mSOD^{wt})
**hSOD1G93A FFPE (n=5)**	120 ± 3	F	[b]Transgenic (hSOD1^{G93A})

[a]B = brain; SC = spinal cord. [b]Formalin-fixed, paraffin embedded (FFPE) tissue.

Supplementary Note 1: Identification and characterization of protein complexes.

A summary of the hSOD1^{G93A} protein complexes and their components detected in this work is included in *Figure S2*. Characterization of the SOD1 protein complexes observed in native MSI experiments was performed by top-down proteomics, with either nanospray-desorption electrospray ionization (nano-DESI[1]) and native-like solvent[2], or liquid extraction surface analysis (LESA[3]) with denaturing solvents as necessary[4]. hSOD1^{G93A} and hSOD1^{wt} complexes were initially identified after spectral deconvolution (*Figure S3 & S4*) by their intact molecular weights (*Table S2*). The stoichiometry of the complexes was confirmed by gas-phase collisional dissociation (*Figure S5 – S7*); dimers were confirmed if monomers were detected as product ions. Further collisional and electron-mediated activation of the monomer product ions revealed protein sequence information, including that hSOD1^{G93A} was N-acetylated (*Figure S8-S9, Table S3-S4*) and that some portion of the metal-deficient hSOD1^{G93A} dimers featured the intact intramolecular disulfide bond (*Figure S10, Table S5*) [5]. To confirm that monomers detected directly from the tissue were endogenous and not the product of collisional activation within the mass spectrometer, the SIM-mode mass spectra were compared with HCD MS^2 spectra of the dimers (*Figure S11*). Collisional activation is accompanied by characteristic metal ion rearrangement which was not observed in the SIM-mode mass spectra confirming that the monomers were present in the tissue. (Note, it is possible that in-solution dissociation of dimers may occur after extraction and prior to ionization; however, the timescale for this is < 1 s). Proton transfer charge reduction (PTCR) MS of the 12+ dimers confirmed that there was no overlap with endogenous monomers in the 6+ charge state with dimers in the 12+ charge state (*Figure S12*). Glutathionylated species were identified by their intact mass (*Figure S3*, *Table S2*), subunit pattern (monomer signals with additional MW ~305 Da, *Figure S7*) and sequence information (*Figure S13, Table S6*). Signals for protein-metal complexes were compared to calculated signals for non-specific binding of salt adducts, for which evidence was not found (e.g., xNa^+, *Figure S14*).

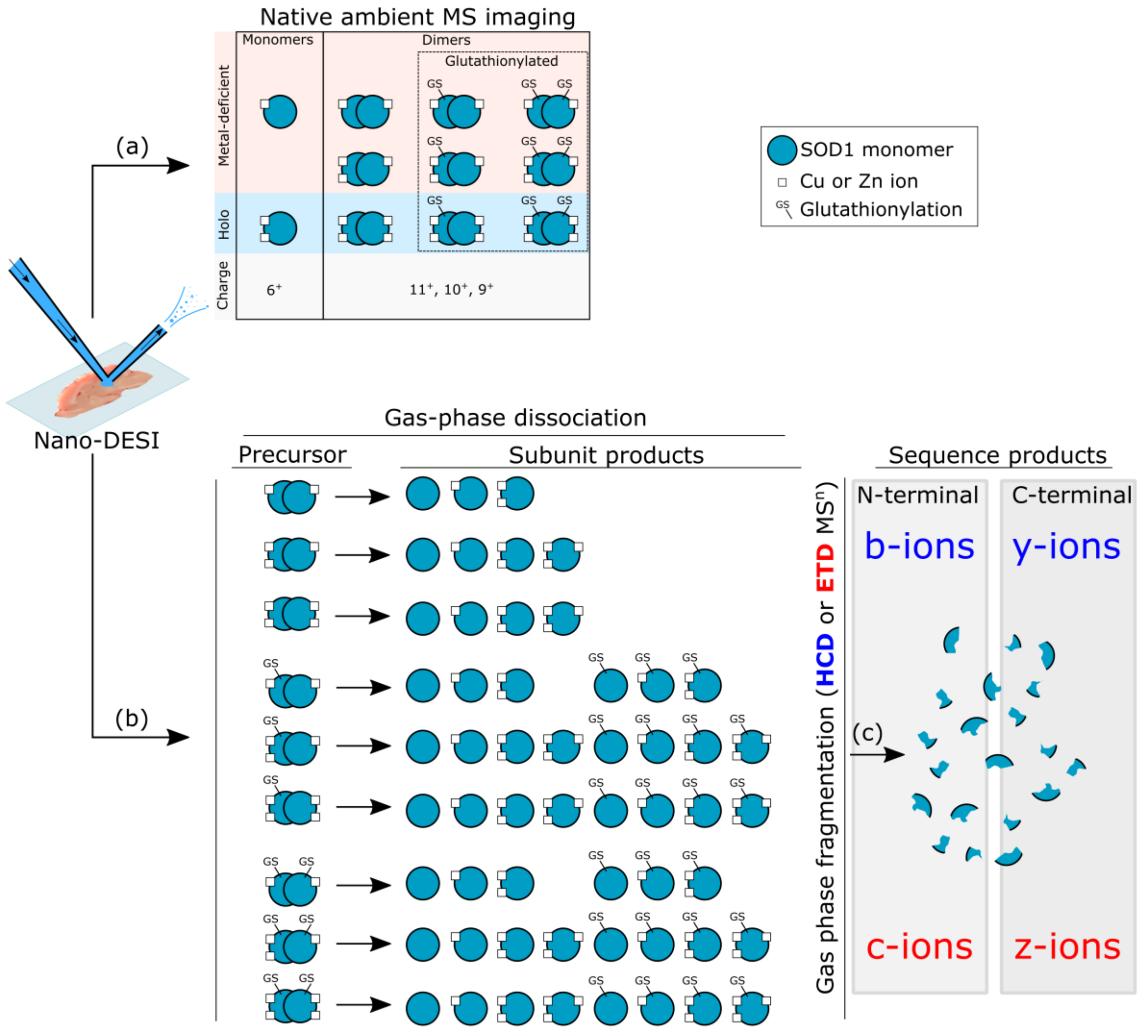

Figure S2: (a) Protein complexes detected by nano-DESI-SIM MS imaging under non-denaturing conditions within the m/z window m/z 3197 +/- 625. (Note that other charge states of these molecules may be detected in low abundance outside of the SIM window). (b) The protein complexes detected were characterised by gas-phase dissociation, i.e., were subjected to collisional activation and dissociated to intact subunit ions via higher-energy collision dissociation (HCD). Metal ions can rearrange to form non-biological complexes (e.g., monomers bound to 3 metal ions) during this process. Some covalent PTMs may also be retained on the complexes (e.g., S-glutathionylation). (c) Further characterisation (protein sequencing) is achieved by gas-phase fragmentation of the complexes and subunits. Sequence ions can be generated by HCD (b and y ions) or electron transfer dissociation (ETD; c and z ions).

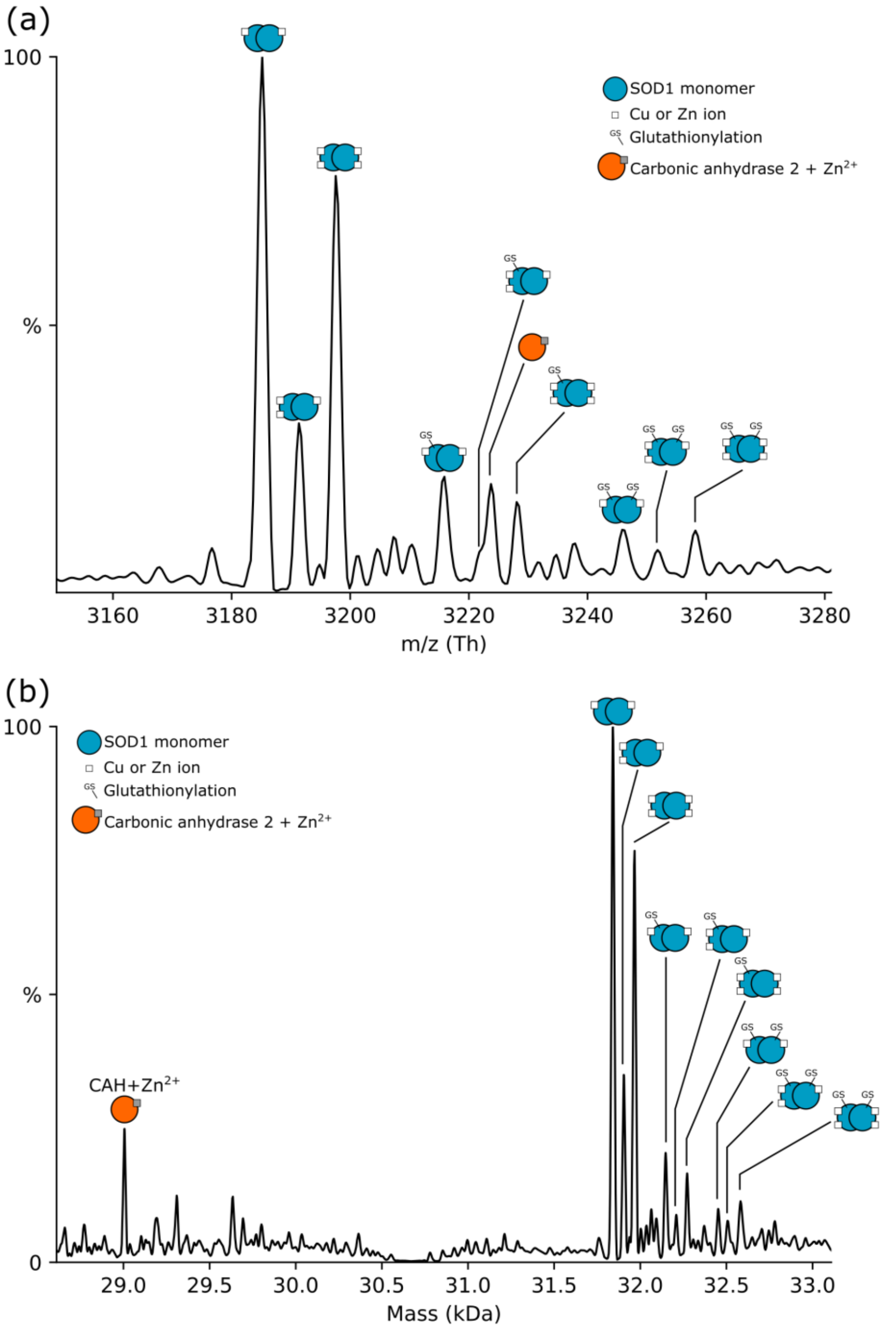

Figure S3: nano-DESI-SIM mass spectrum (m/z 3197 ± 625) from hSOD1^{G93A} transgenic mouse brain. (a) signals for the 10+ dimers and zinc-bound carbonic anhydrase 2 (9+). hSOD1^{G93A} may be glutathionylated. Spectrum acquired at an orbitrap resolution of 7500 (at m/z 200) and is averaged from 3830 scans in the brainstem. (b) deconvoluted mass spectrum showing the nine monitored hSOD1^{G93A} dimer species and [carbonic anhydrase+Zn^{2+}] complex. Deconvoluted from multiple detected charge states with UniDec [6].

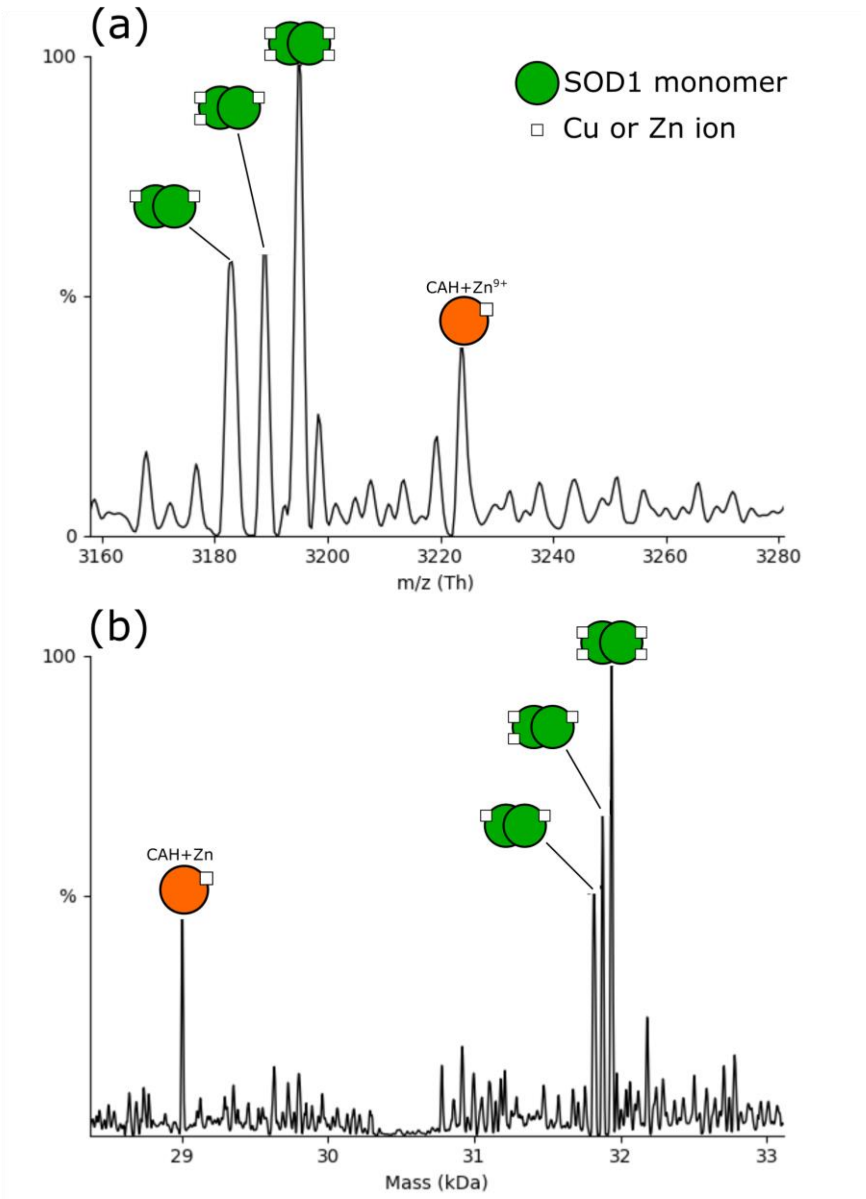

Figure S4: nano-DESI-SIM mass spectrum (m/z 3197 ± 625) from hSOD1^{wt} transgenic mouse brain. (a) signals for the 10+ hSOD1^{wt} dimers and zinc-bound carbonic anhydrase 2 (9+). Spectrum acquired at an orbitrap resolution of 7500 (at m/z 200) and is averaged from 190 scans in the hippocampus. (b) deconvoluted mass spectrum showing the three monitored hSOD1^{wt} dimer species and [carbonic anhydrase+Zn^{2+}] complex. Deconvoluted from multiple detected charge states with UniDec [6].

Table S2: Calculated and measured molecular weights (MW) for hSOD1G93A and hSOD1wt dimer complexes.

Symbol	Metal ions	Molecular formula[a]	Calculated MW (dimer)/ Da	Measured MW (dimer)/ Da
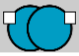	2	$C_{1364}H_{2168}N_{406}O_{450}S_8Zn_2$	31842	31841
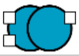	3	$C_{1364}H_{2167}N_{406}O_{450}S_8Zn_2Cu$	31904	31905
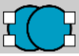	4	$C_{1364}H_{2166}N_{406}O_{450}S_8Zn_2Cu_2$	31967	31966
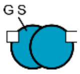	2	$C_{1374}H_{2183}N_{409}O_{456}S_9Zn_2$	32147	32148
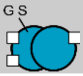	3	$C_{1374}H_{21}82N_{409}O_{456}S_9Zn_2Cu$	32210	32210
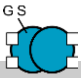	4	$C_{1374}H_{2181}N_{409}O_{456}S_9Zn_2Cu_2$	32272	32272
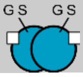	2	$C_{1384}H_{2198}N_{412}O_{462}S_{10}Zn_2$	32452	32453
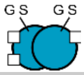	3	$C_{1384}H_{2197}N_{412}O_{462}S_{10}Zn_2Cu$	32515	32505
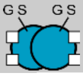	4	$C_{1384}H_{2196}N_{412}O_{462}S_{10}Zn_2Cu_2$	32577	32581
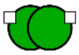	2	$C_{1362}H_{2170}N_{406}O_{450}S_8Zn_2$	31820	31820
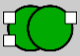	3	$C_{1362}H_{2169}N_{406}O_{450}S_8Zn_2Cu$	31882	31879
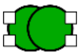	4	$C_{1362}H_{2168}N_{406}O_{450}S_8Zn_2Cu_2$	31945	31940

[a] Assumed oxidation states: Zn^{2+}, Cu^{+}.[7]

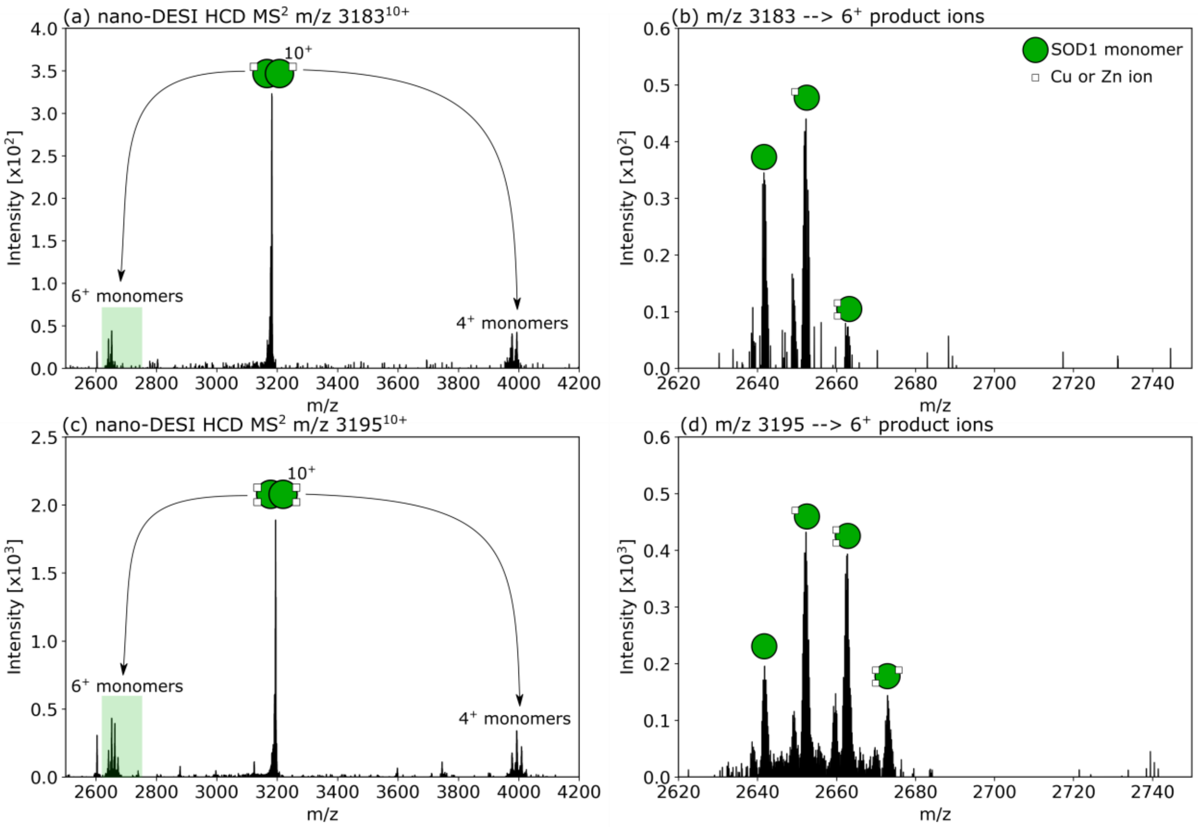

Figure S5: nano-DESI-HCD MS^2 spectra showing gas phase dissociation of hSOD1^{wt} dimers extracted from the hippocampus. (a) 10+ charge state of the 2-metal dimer. (b) expanded region shows 6+ product ions bind a maximum of two metal ions after rearrangement during HCD. (c) 10+ charge state of the 4-metal dimer. (d) expanded region shows 6+ product ions bind 0-3 metals.

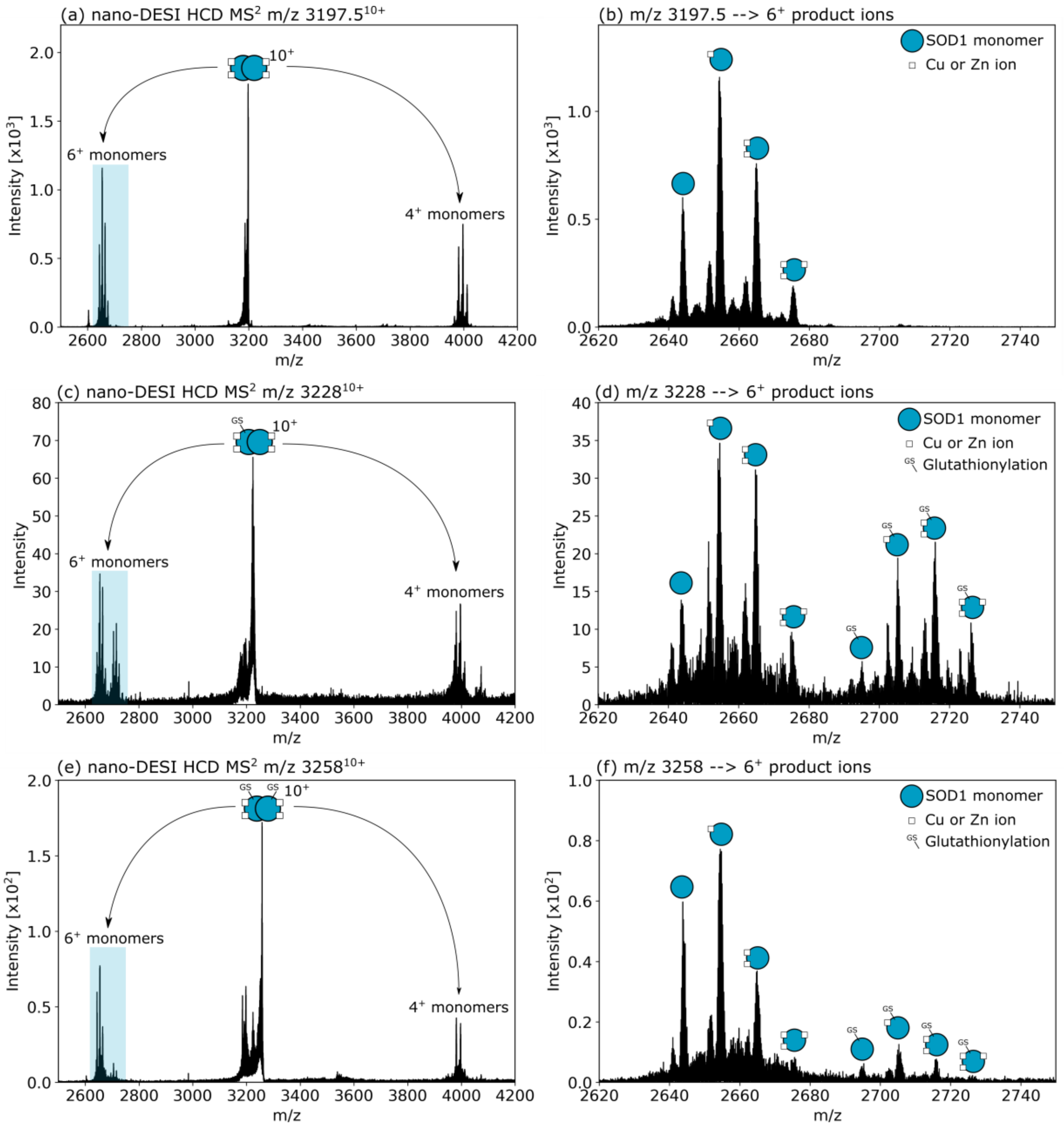

Figure S6: Nano-DESI HCD MS² mass spectra of hSOD1^{G93A} dimers incorporating four metal ions and unmodified and glutathionylated polypeptide chains. (a) m/z 3197.5, corresponding to a dimer containing two unmodified subunits. (b) expanded m/z region of HCD MS² spectrum showing the 6+ product ions corresponding to unmodified monomers in various metalated states. (c) m/z 3228, corresponding to a dimer containing one glutathionylated subunit. (d) expanded m/z region of HCD MS² spectrum showing the 6+ product ions corresponding to unmodified and glutathionylated monomers in various metalated states. (e) m/z 3258, corresponding to a dimer containing two glutathionylated subunits. (f) expanded m/z region of HCD MS² spectrum showing the 6+ product ions corresponding to unmodified and glutathionylated monomers in various metalated states. Orbitrap resolution = 240,000 at m/z 200. Peaks to the immediate left of the dimer peaks correspond to loss of glutathione, water and/or metal ions without dimer dissociation. Dissociation of glutathione and metal ion rearrangement amongst monomers occurs during collisional activation.

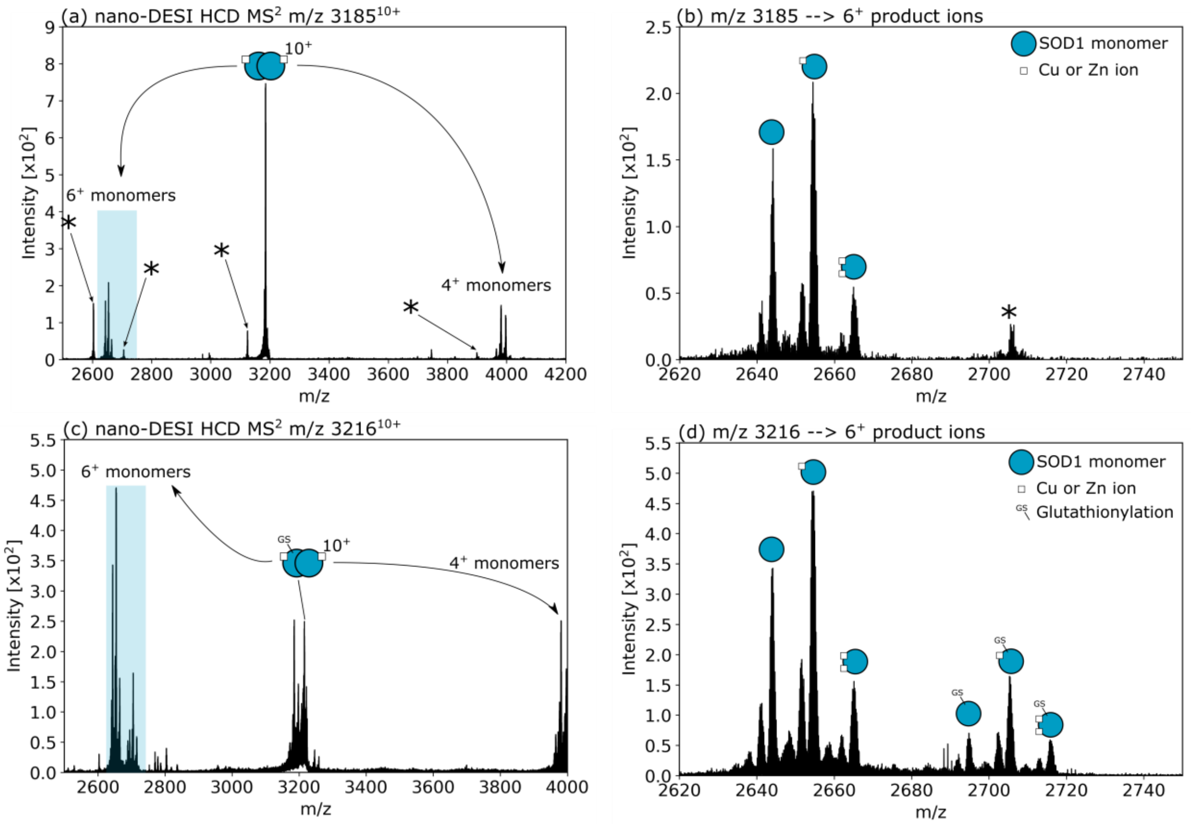

Figure S7: Nano-DESI HCD MS^2 mass spectra of hSOD1^{G93A} dimers incorporating two metal ions and unmodified and glutathionylated polypeptide chains. (a) m/z 3185, corresponding to a dimer containing two unmodified subunits. ***** The asterisk indicates hemoglobin related product ions owing to overlap in the isolation window (b) an expanded HCD MS^2 spectrum of the 6+ product ions shows signals for unmodified monomers in various metalated states. (c) m/z 3216, corresponding to a dimer containing one glutathionylated subunit. (d) an expanded HCD MS^2 spectrum of the 6+ product ions shows signals for unmodified and glutathionylated monomers in various metalated states. Orbitrap resolution = 240,000 at m/z 200. Peaks to the immediate left of the dimer peaks correspond to loss of glutathione, water and/or metal ions without dimer dissociation. Dissociation of glutathione and metal ion rearrangement amongst monomers occurs during collisional activation.

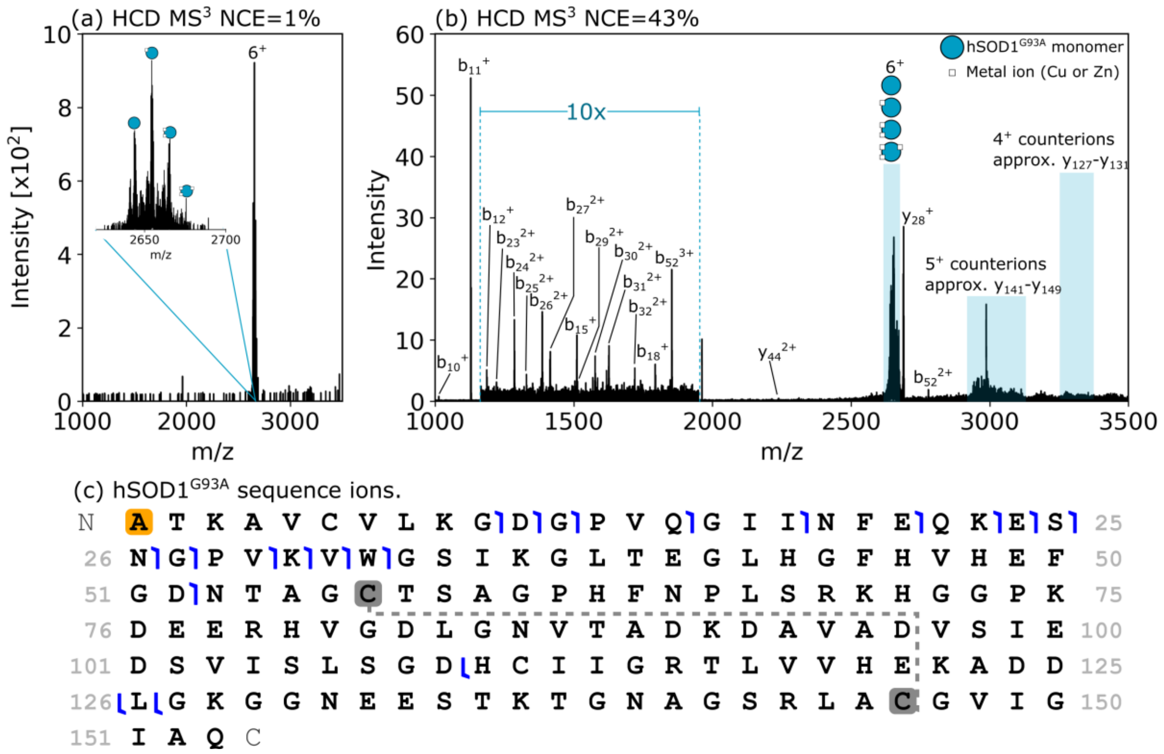

Figure S8: nano-DESI-HCD MS^3 spectra obtained under non-denaturing conditions for m/z 3198.5±10 → m/z 2664.0±40 → product ions. (a) The MS^3 isolation window contained 6$^+$ hSOD1^{G93A} monomers in multiple metal-bound states (MS^2 NCE: 40%; MS^3 NCE: 1%). (b) MS^3 product ion spectrum showing sequence ion signals (MS^2 NCE: 40%; MS^3 NCE: 43%). (c) MS^3 product ions mapped to the sequence of hSOD1^{G93A}. The orange highlight indicates N-terminal acetylation. The grey highlight and connecting line indicate the disulfide bridge.

Table S3: sequence ions for HCD MS^3 analysis of hSOD1^{G93A}.

Ion	Calculated Mass (Da)	Observed Mass (Da)	Mass Difference (ppm)
**b10**	1012.5634	1012.5656	2.2
**b11**	1127.5903	1127.5998	8.5
**b12**	1184.6118	1184.6136	1.5
**b15**	1508.7915	1508.7989	4.9
**b18**	1791.9811	1791.9870	3.3
**b21**	2182.1350	2182.1318	-1.5
**b23**	2438.2998	2438.2826	-7.1
**b24**	2567.3311	2567.3445	5.2
**b25**	2654.3744	2654.3406	-12.7
**b26**	2768.4061	2768.3832	-8.3
**b27**	2825.4276	2825.4061	-7.6
**b29**	3021.5600	3021.5978	12.5
**b30**	3149.6437	3149.6060	-12.0
**b31**	3248.7121	3248.6920	-6.2
**b32**	3434.8026	3434.7954	-2.1
**b52**	5552.8196	5552.7672	-9.4
**y27**	2572.2666	2572.2381	-11.1
**y28**	2686.3582	2686.3086	-18.5
**y44**	4473.2730	4473.2249	-10.7

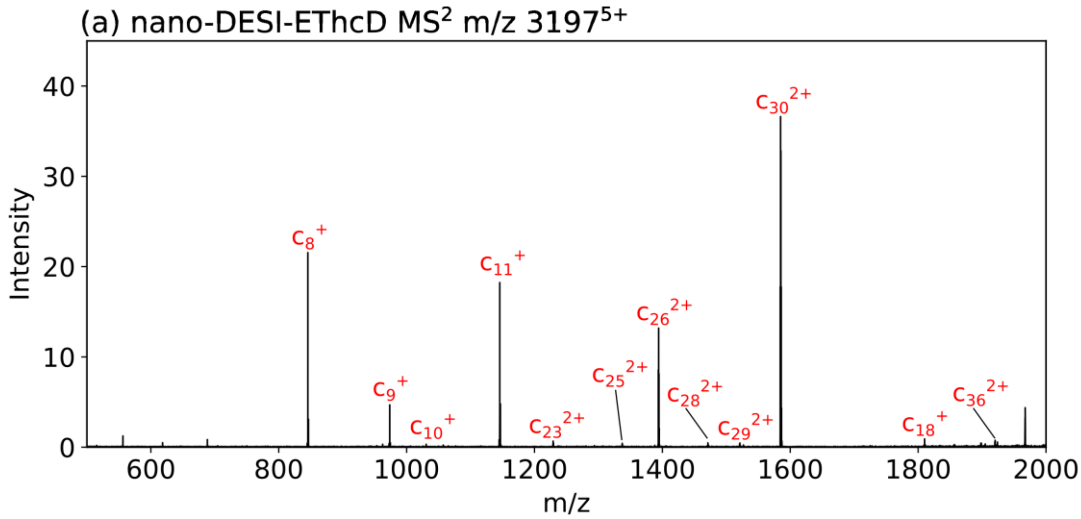

(b) hSOD1^{G93A} ETD sequence ions.

N A T K A V C V L K G D G P V Q G I I N F E Q K E S 25
26 N G P V K V W G S I K G L T E G L H G F H V H E F 50
51 G D N T A G C T S A G P H F N P L S R K H G G P K 75
76 D E E R H V G D L G N V T A D K D A V A D V S I E 100
101 D S V I S L S G D H C I I G R T L V V H E K A D D 125
126 L G K G G N E E S T K T G N A G S R L A C G V I G 150
151 I A Q C

Figure S9: (a) nano-DESI-EThcD MS^2 of hSOD1^{G93A} generated N-terminal c-ions only. (b) Sequence of hSOD1^{G93A} with detected c-ions labelled. Red highlighted N-terminus indicates acetylation. ETD reaction time; 10-18 ms. Normalized supplemental collisional activation; 10-12%. Note: ETD product ions >m/z 2000 were not detectable.

Table S4: sequence ions for EThcD MS^2 analysis of hSOD^{G93A}.

Ion	Calculated Mass (Da)	Observed Mass (Da)	Mass Difference (ppm)
**c8**	844.4838	844.4847	1.1
**c9**	972.5788	972.5813	2.6
**c10**	1029.6008	1029.6008	0.0
**c11**	1144.6272	1144.6273	0.2
**c18**	1809.0185	1809.0143	-2.3
**c23**	2455.3254	2455.3121	-5.4
**c25**	2671.4000	2671.4070	2.6
**c26**	2785.4430	2785.4268	-5.8
**c28**	2939.5172	2939.5077	-3.2
**c29**	3038.5856	3038.5883	0.9
**c30**	3166.6806	3166.6699	-3.4
**c36**	3837.0608	3837.0347	-6.8

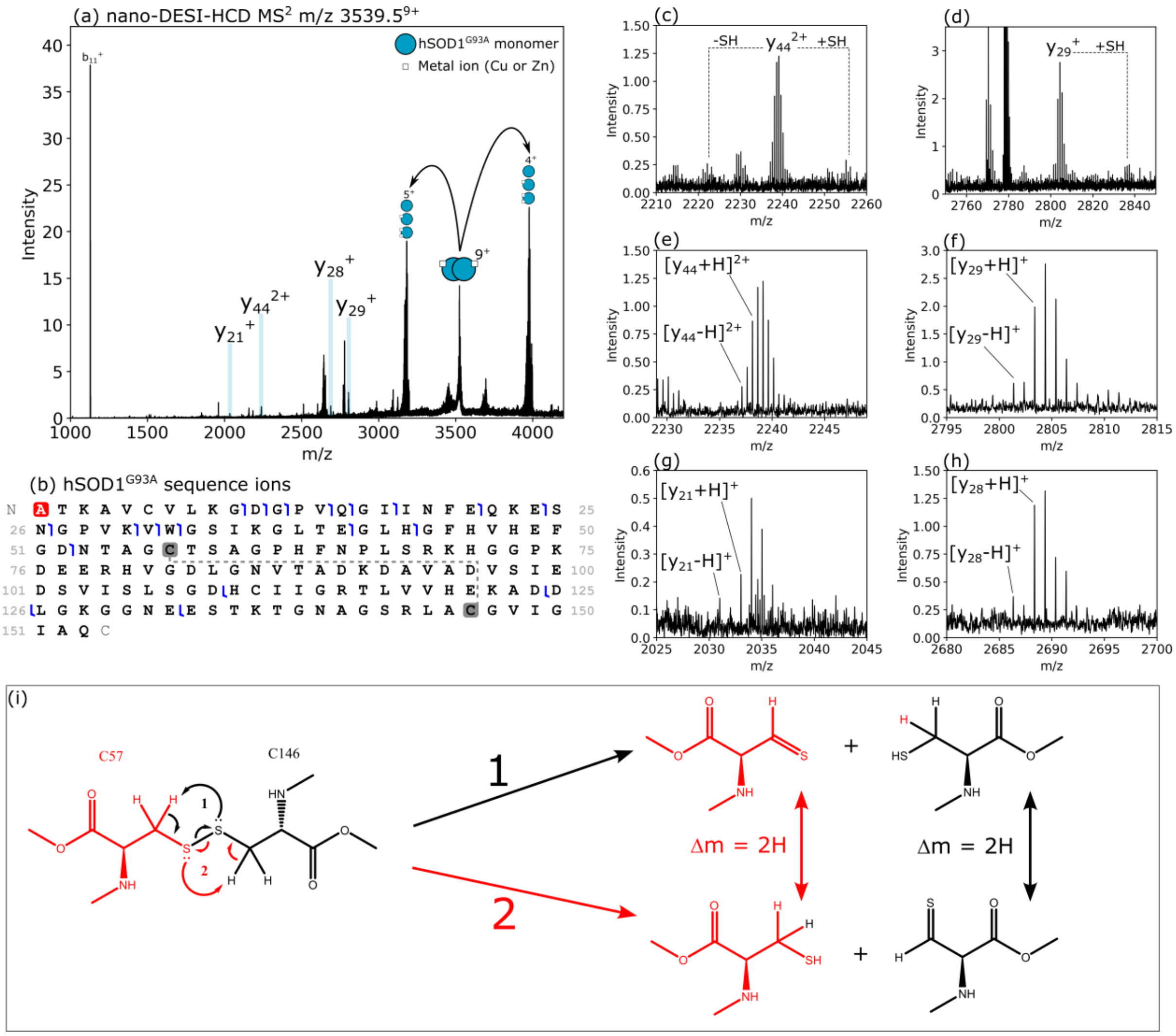

Figure S10: (a) nano-DESI-HCD MS[2] spectrum of hSOD1[G93A] dimers bound to two metal ions. 9+ charge state was chosen to avoid overlap with monomer signals. (b) sequence ions detected by fragmentation of the dimers. (c-h) y-ions y_{44}^{2+}, y_{29}^{+}, y_{21}^{+} and y_{28}^{+} show evidence of disulfide bond cleavage by gas phase collisional activation. (i) scheme showing the formation of y_n-H and y_n+H ions. For detailed mechanisms, see [5].

Table S5: sequence ions for hSOD1^{G93A} with an intact intramolecular disulfide bond from metal-deficient dimers.

Ion	Calculated mass (Da)	Observed Mass (Da)	Mass Difference (ppm)
**b10**	1012.5739	1012.5684	-5.4
**b11**	1127.6009	1127.5946	-5.5
**b12**	1184.6223	1184.6103	-10.2
**b14**	1380.7508	1380.7394	-8.3
**b15**	1508.8021	1508.7913	-7.1
**b17**	1678.9149	1678.9016	-7.9
**b21**	2182.1456	2182.1190	-12.2
**b24**	2567.3417	2567.3232	-7.2
**b24**	2567.3564	2567.3378	-7.2
**b26**	2768.4167	2768.3803	-13.1
**b30**	3149.6543	3149.6309	-7.4
**b31**	3248.7227	3248.6997	-7.1
**b32**	3434.8020	3434.7573	-13.0
**b40**	4220.2303	4220.1972	-7.8
**b43**	4527.3947	4527.3594	-7.8
**b52**	5552.8302	5552.7799	-9.1
**y21**	2030.0288	2030.0073	-10.6
**y28**	2685.3577	2685.3302	-10.2
**y29**	2801.3851	2801.3433	-14.9
**y44**	4472.2872	4472.2806	-1.5

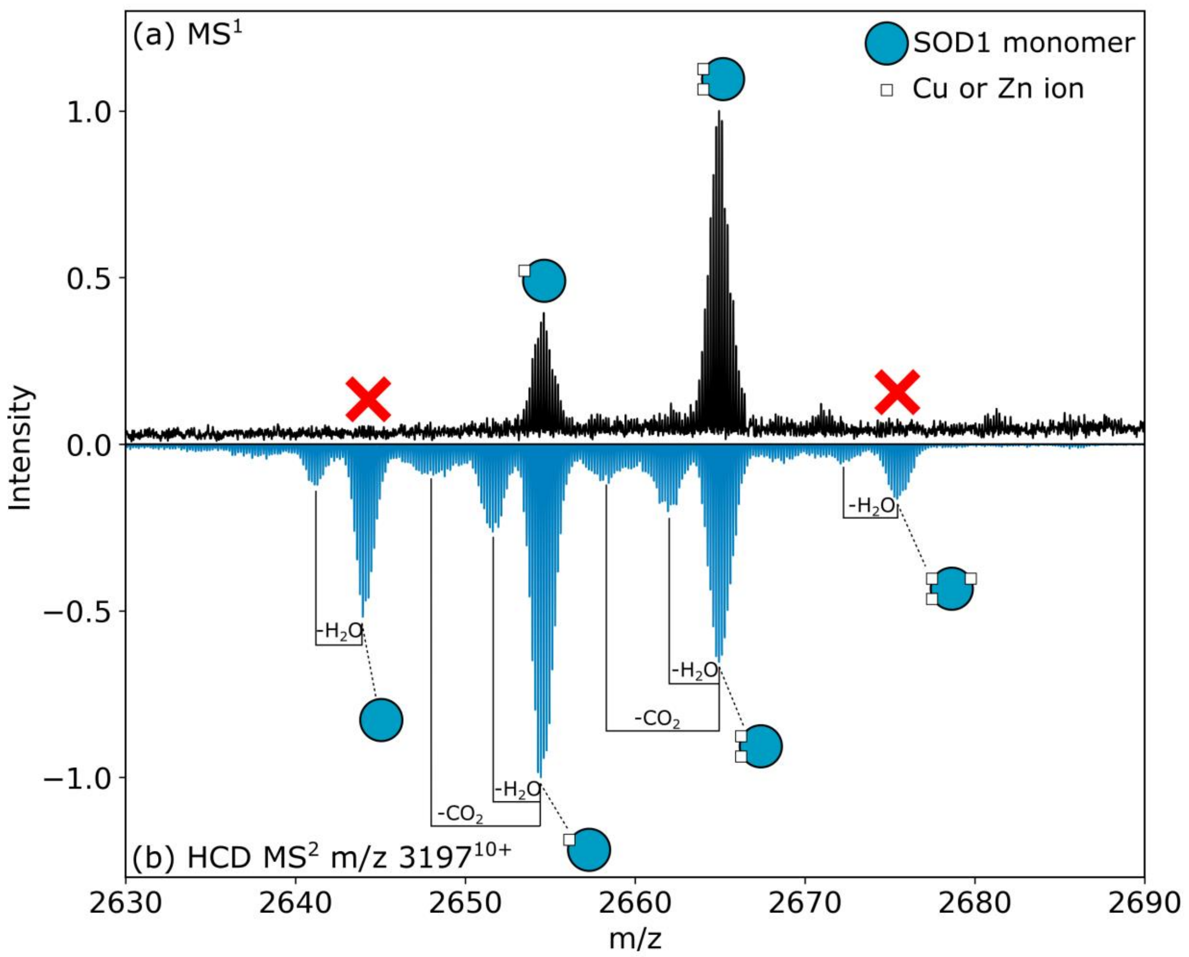

Figure S11: (a) nano-DESI MS spectrum recorded from the brainstem under experimental conditions used for imaging. hSOD^{G93A} monomer signals in the brainstem (6+ charge state) were detected bound only to 1 metal ion or 2 metal ions. (b) Nano-DESI-HCD MS^2 spectrum of hSOD1^{G93A} dimers (m/z 3197^{10+}±4). Upon collisional activation (normalised collision energy > 30% with the IRM pressure set to 20 mTorr) metal ions rearrange to form complexes without biological origin i.e. monomers bound to 3 metal ions. Complete dissociation of metal ions from the monomer also occurs leaving apo-hSOD1^{G93A}. Collisional activation also caused neutral loss fragmentation of H_2O and CO_2 molecules. Absence of signals for the proteoforms and neutral loss products detected in mass spectrum (b) in mass spectrum (a) indicates that monomers in the MS images were formed endogenously.

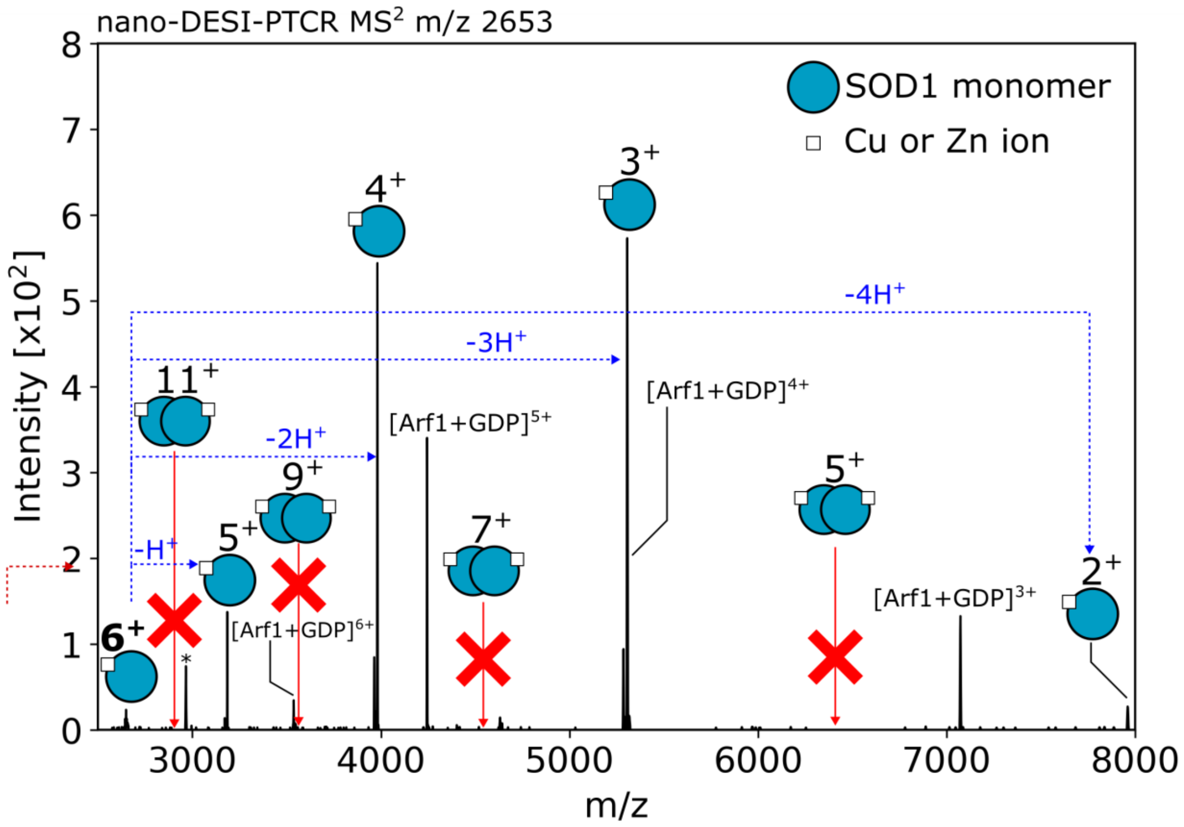

Figure S12: Nano-DESI-PTCR MS^2 of m/z 2653±10. Hypothetically, monomer signals and dimer signals (1 metal ion, 6+ charge state; 2 metal ions, 12+ charge state, respectively) could overlap at this m/z. PTCR indicates that there is no overlap: PTCR product ions of dimeric hSOD1^{G93A} with odd numbers of charge were not detected. Note: signals for [Arf1+GDP]$^{n+}$ complex are the result of charge reduction of [Arf1+GDP]$^{7+}$ within the isolation window, approx. m/z 2651. *indicates instrument-specific electrical noise.

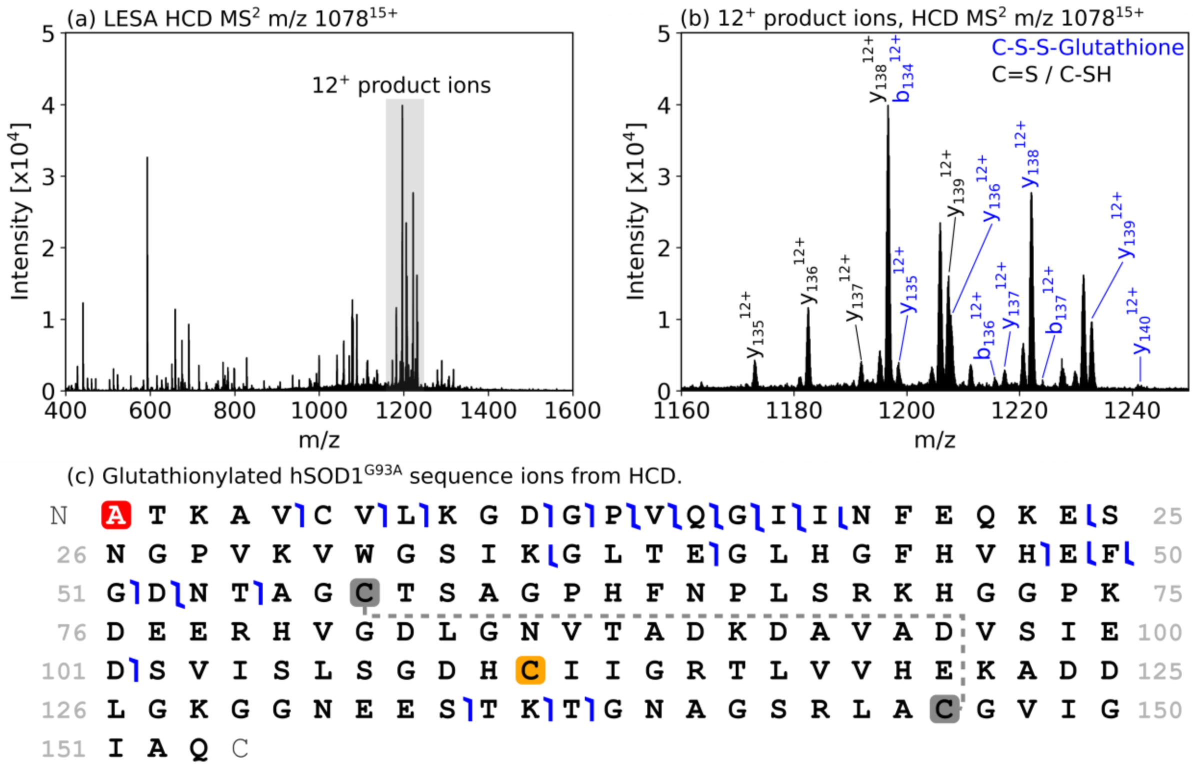

Figure S13: (a) LESA-HCD MS² spectrum of glutathionylated hSOD1^{G93A} monomers (m/z 1078^{15+}) under denaturing solvent conditions where metal ions are dissociated in-solution. (b) amongst the 12+ product ions, b and y ions retaining glutathione were detected. (c) HCD product ions (i.e. b, y) are labelled on the GS-hSOD1^{G93A} sequence.

Table S6: HCD product ions from m/z 1078^{15+}

Ion	Theoretical Mass (Da)	Observed Mass (Da)	Mass Difference (ppm)
**b5**	512.2958	512.2960	0.2
**b7**	714.3734	714.3738	0.5
**b8**	827.4575	827.4581	0.7
**b11**	1127.6009	1127.6035	2.3
**b12**	1184.6223	1184.6235	1.0
**b13**	1281.6751	1281.6760	0.7
**b14**	1380.7435	1380.7430	-0.4
**b15**	1508.8021	1508.7996	-1.7
**b16**	1565.8235	1565.8231	-0.3
**b17**	1678.9076	1678.9067	-0.5
**b40**	4220.2303	4220.1984	-7.6
**b48**	5104.6708	5104.6342	-7.2
**b51**	5437.7118	5437.8048	17.1
**b52**	5552.8302	5552.8206	-1.7
**b54**	5767.9208	5767.8945	-4.6
**b101**	10616.2378	10616.2140	-2.2
**b134**	14337.9834	14338.0549	5.0
**b136**	14567.1252	14567.1396	1.0
**b137**	14668.1736	14668.1244	-3.4
**y101**	10599.0983	10599.1466	4.6
**y117**	12331.8945	12331.9278	2.7
**y129**	13584.6320	13584.5870	-3.3
**y135**	14359.9380	14359.9824	3.1
**y136**	14473.0224	14473.0500	1.9
**y137**	14586.1056	14586.1188	0.9
**y138**	14643.1272	14643.1512	1.6
**y139**	14771.1864	14771.2044	1.2
**y140**	14870.2548	14870.2728	1.2

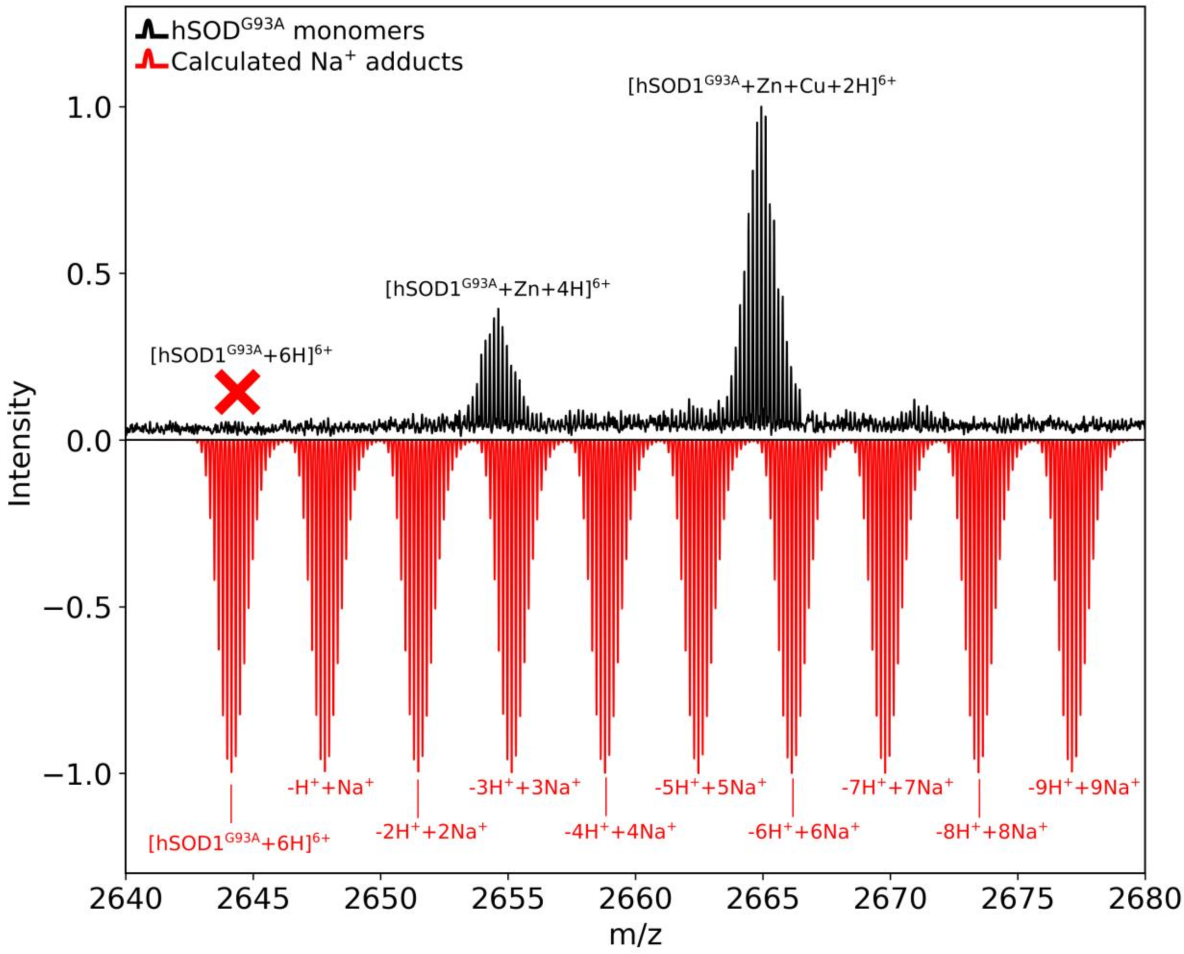

Figure S14: High-resolution (~61,000 FWHM at m/z 2660) nano-DESI mass spectrum for hSOD1^{G93A} monomer ions in the brainstem (black spectrum; 6^+ charge state, 1 and 2 metal ions detected, apo-hSOD1^{G93A} not detected), and a simulated high-resolution (~61,000 FWHM at m/z 2660) mass spectrum (red) for apo-hSOD1^{G93A} and a sodium adduct series. hSOD1^{G93A} sodium adducts do not align with the signals detected from tissue.

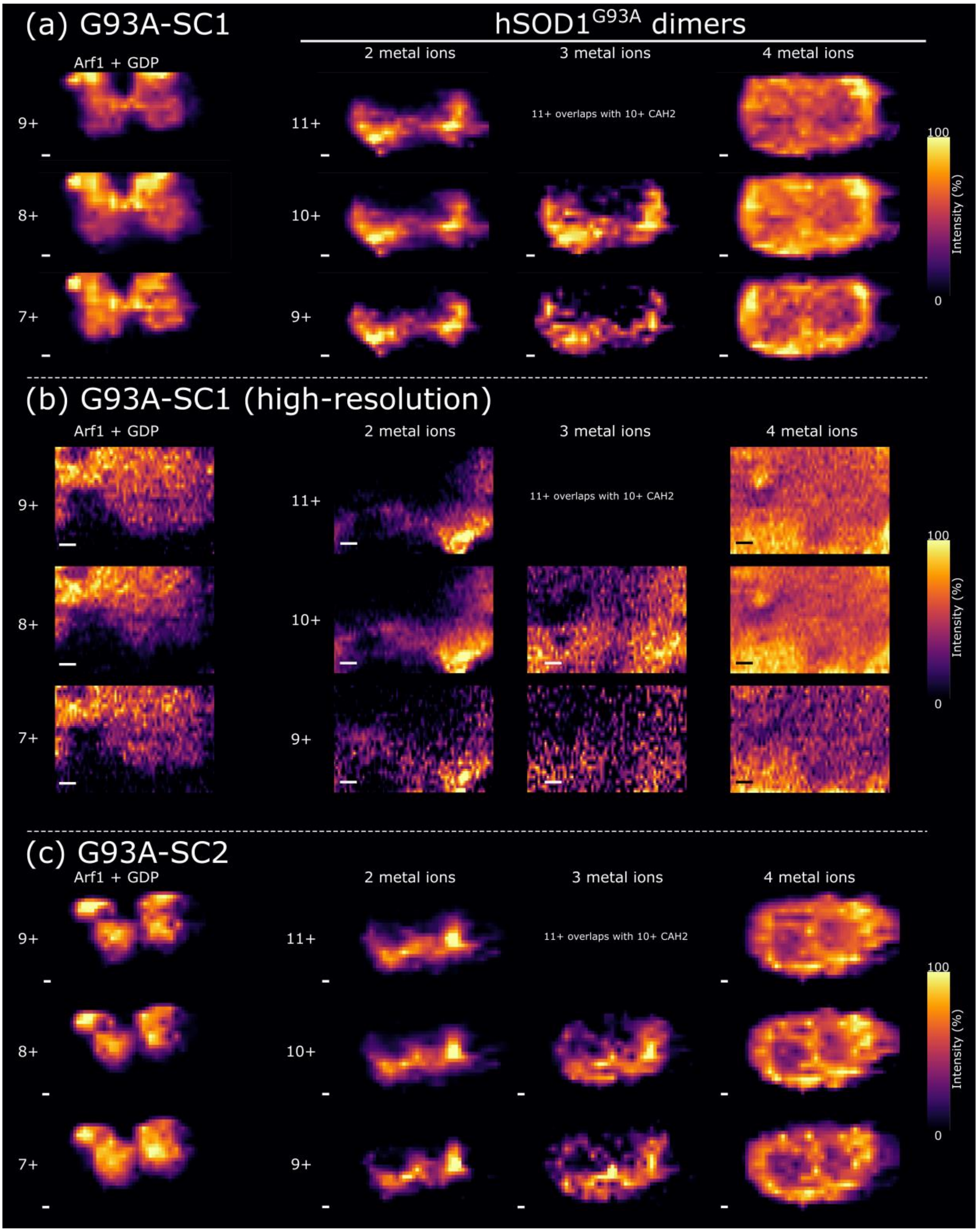

Figure S15: Ion images for proteins in individual charge states for the two hSOD1^{G93A} spinal cords (a) G93A-SC1 and at higher resolution (b). (c) G93A-SC2. Scale bar: 100 μm.

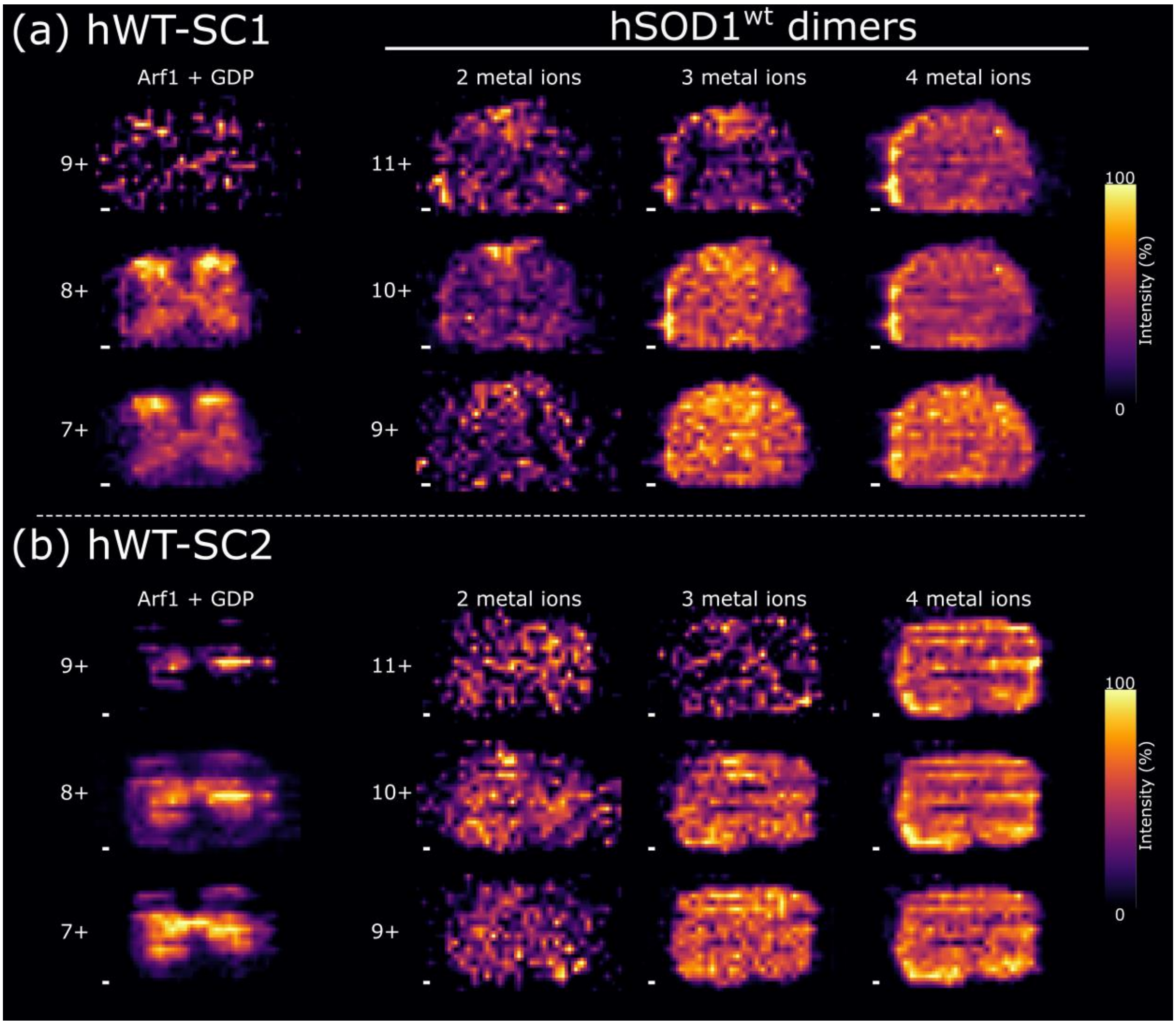

Figure S16: Ion images for proteins in individual charge states for the two hSOD1wt spinal cords (a) hWT-SC1 (b) hWT-SC2. Scale bar: 100 μm.

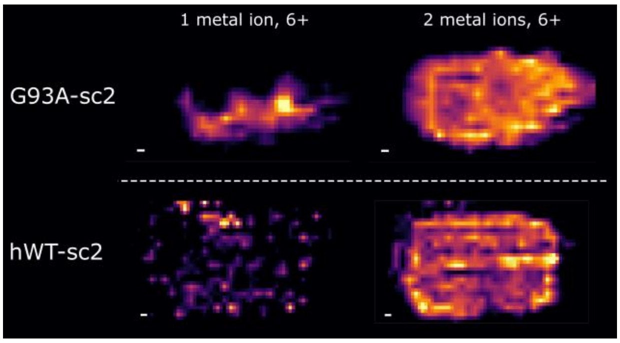

Figure S17: Ion images for hSOD1 monomer (6+ charge state) in biological replicates of spinal cord. Localisation was only detected for the hSOD1G93A monomer with 1 metal ion. Scale bar = 100 μm.

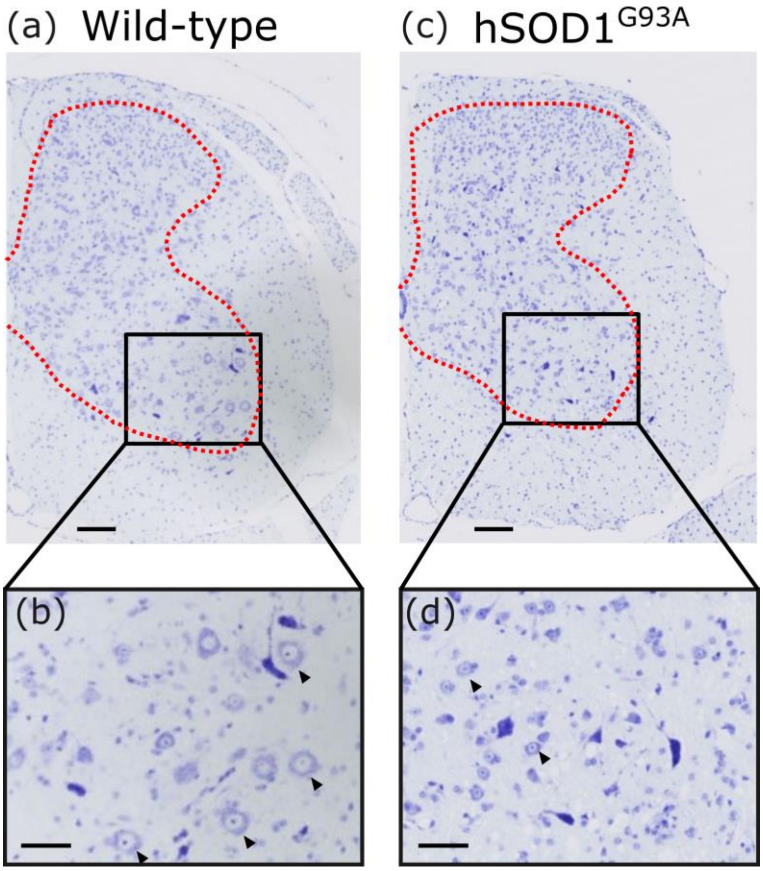

Figure S18: representative FFPE lumbar spinal cord sections from mSOD1^{wt} (representative of 36 sections) and hSOD1^{G93A} (representative of 28 sections) mice at 120 days of age. Motor neurons indicated by black arrows. Scale bar (a, c) = 100 μm, (b, d) 50 μm.

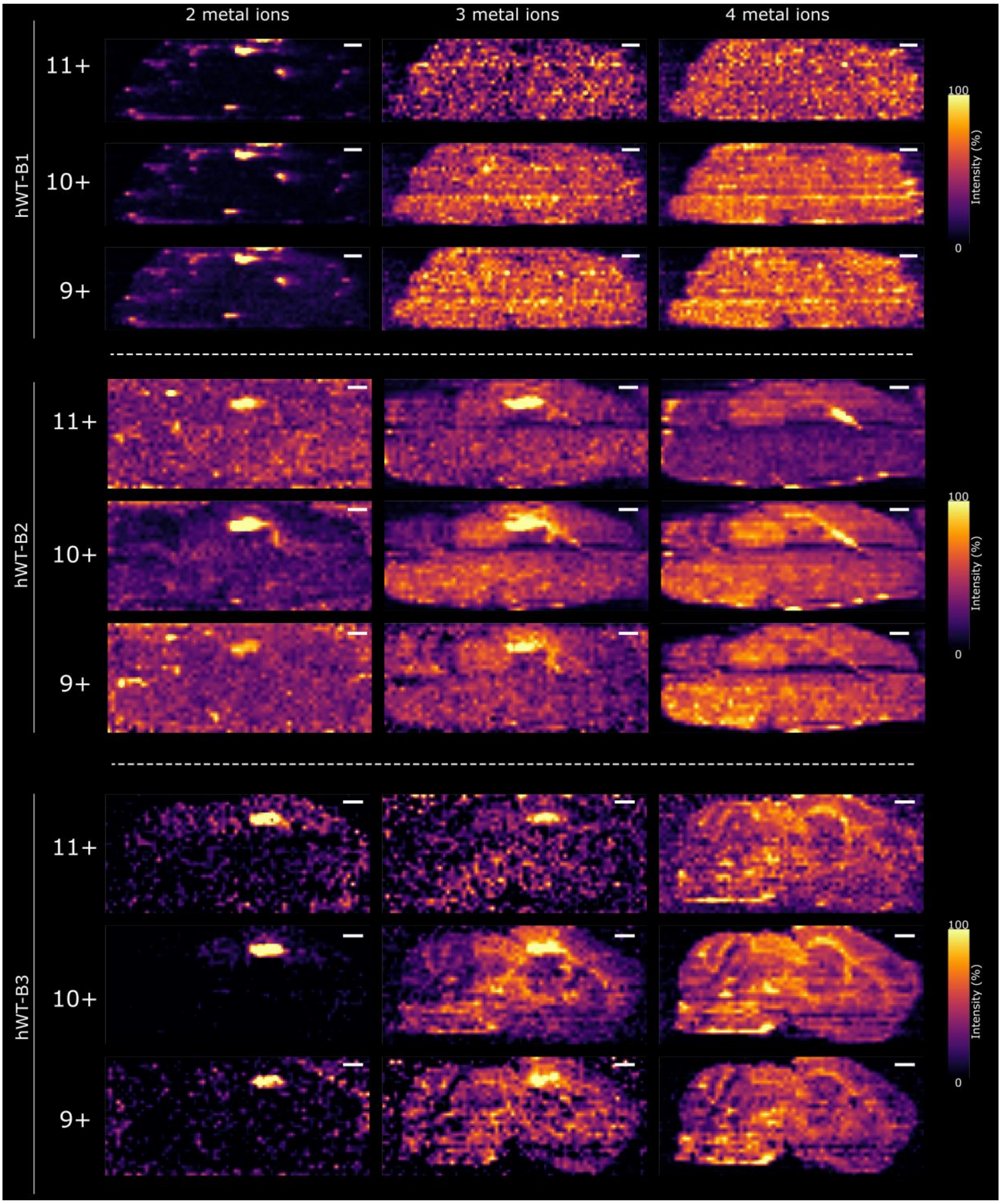

Figure S19: Ion images for hSOD1^{wt} dimers in charge states 11+ - 9+, for the three hSOD1wt mouse brains. Scale bar: 1 mm

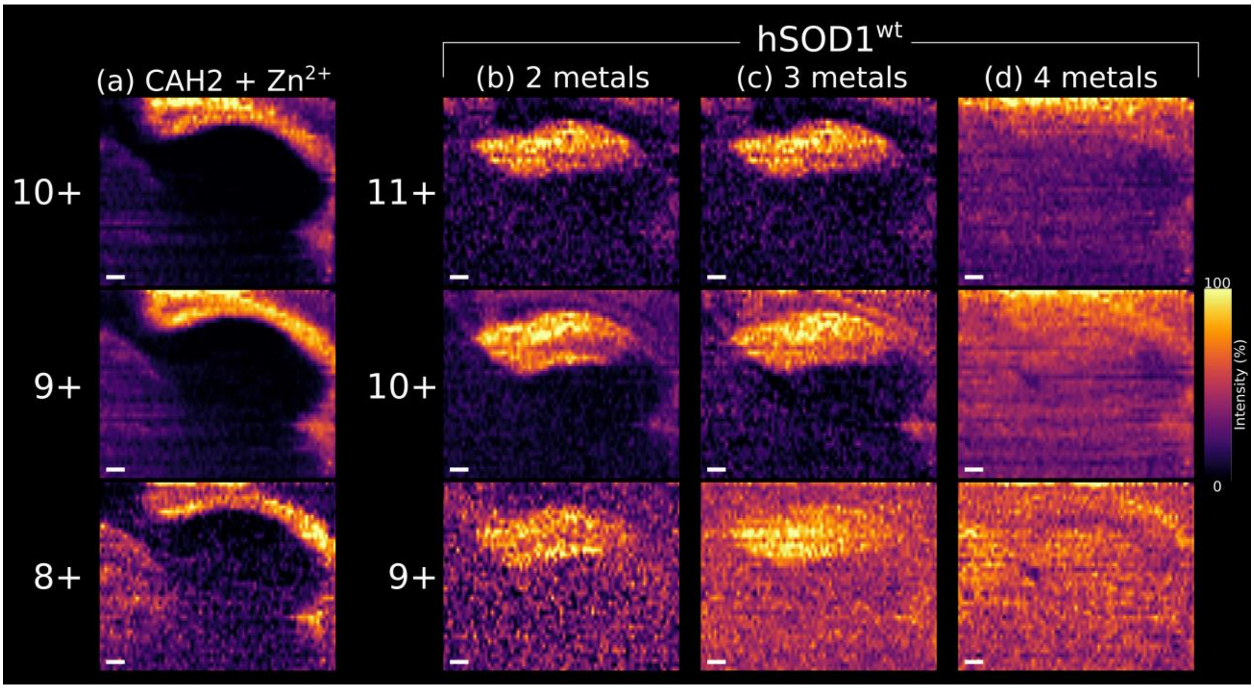

Figure S20: Ion images for each charge state of (a) CAH2 and (b, c, d) three metal-bound states of hSOD1[wt] dimers analyzed from hWT-B2, which comprise high-resolution ion images presented in Figure 3. Scale bar = 200 μm.

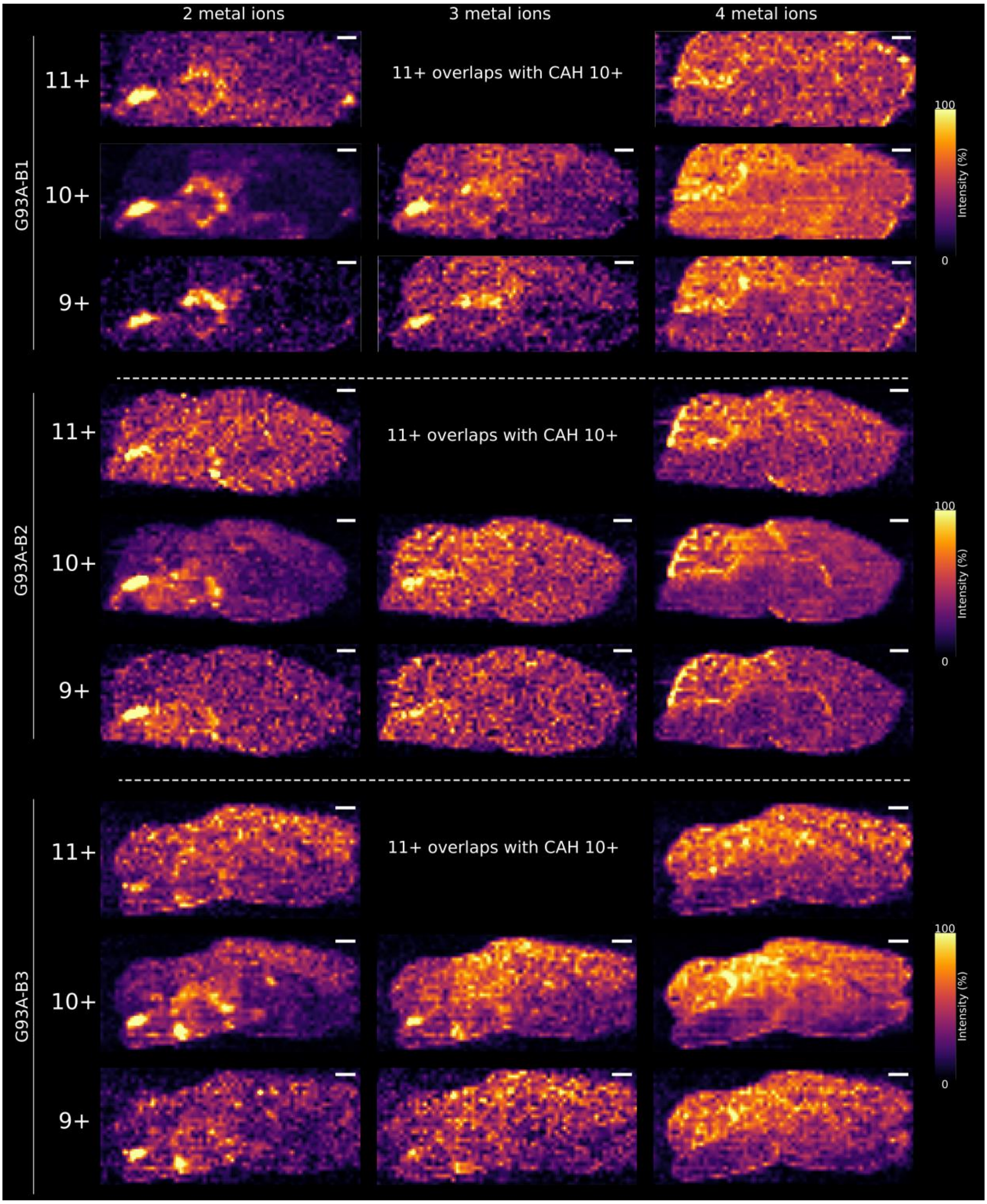

Figure S21: Ion images for hSOD1^{G93A} dimers in charge states 11+ - 9+, for the three G93A mouse brains. Scale bar: 1 mm

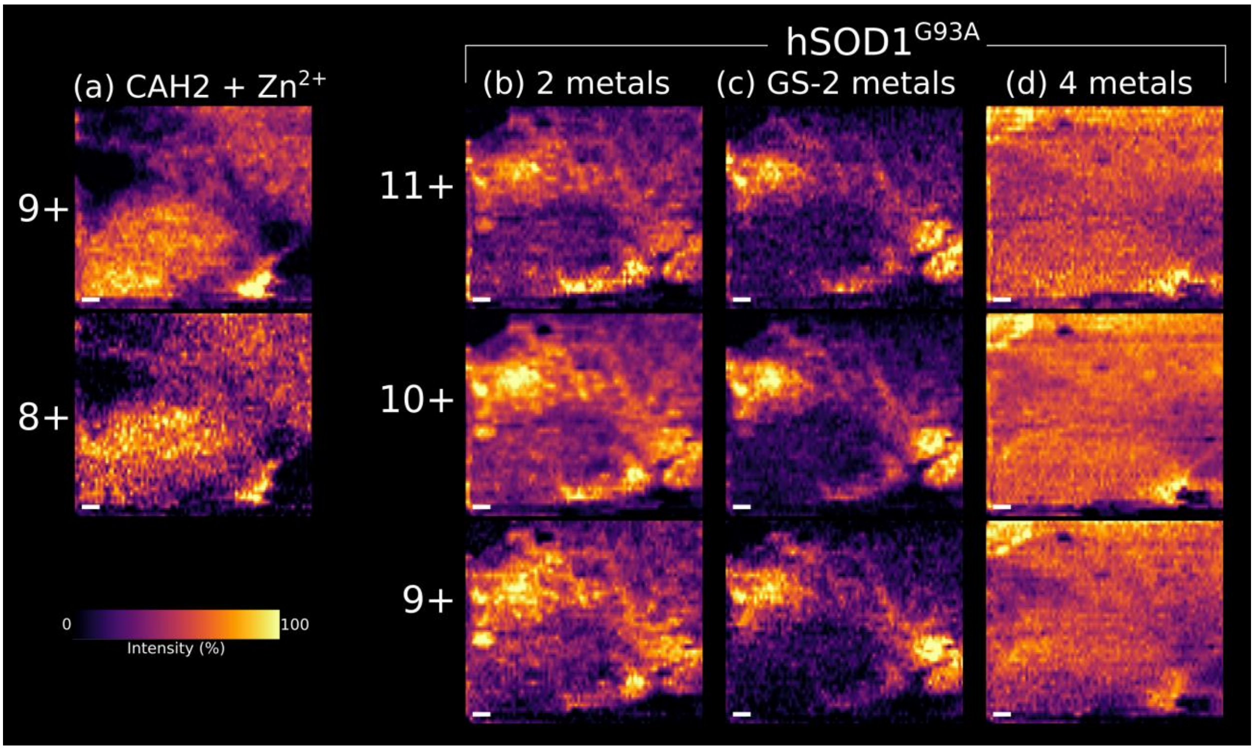

Figure S22: Ion images for each charge state of (a) CAH2 and hSOD1^{G93A} dimers (b; 2 metals, c; 2 metals + 1x glutathionylated subunit, d; 4 metals), which comprise high-resolution ion images presented in Figure 3. Scale bar = 200 μm.

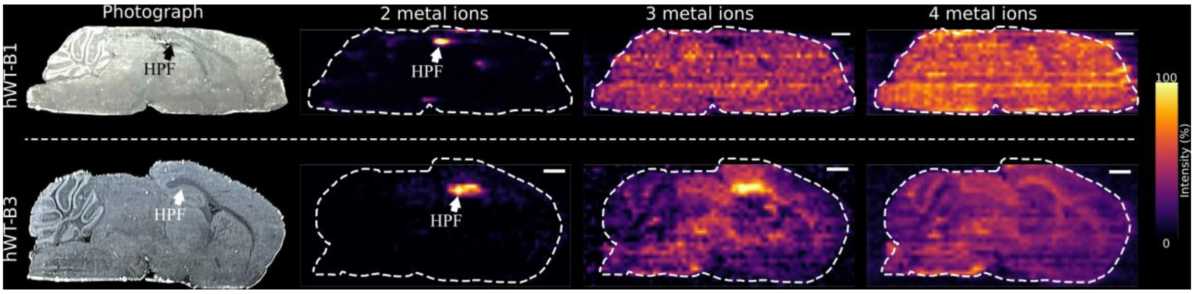

Figure S23: photographs and ion images for hSOD1^{wt} dimers in brains hWT-B1 and hWT-B3. Ion images are composed of signals from 11+ - 9+ charge states. HPF; hippocampal formation.

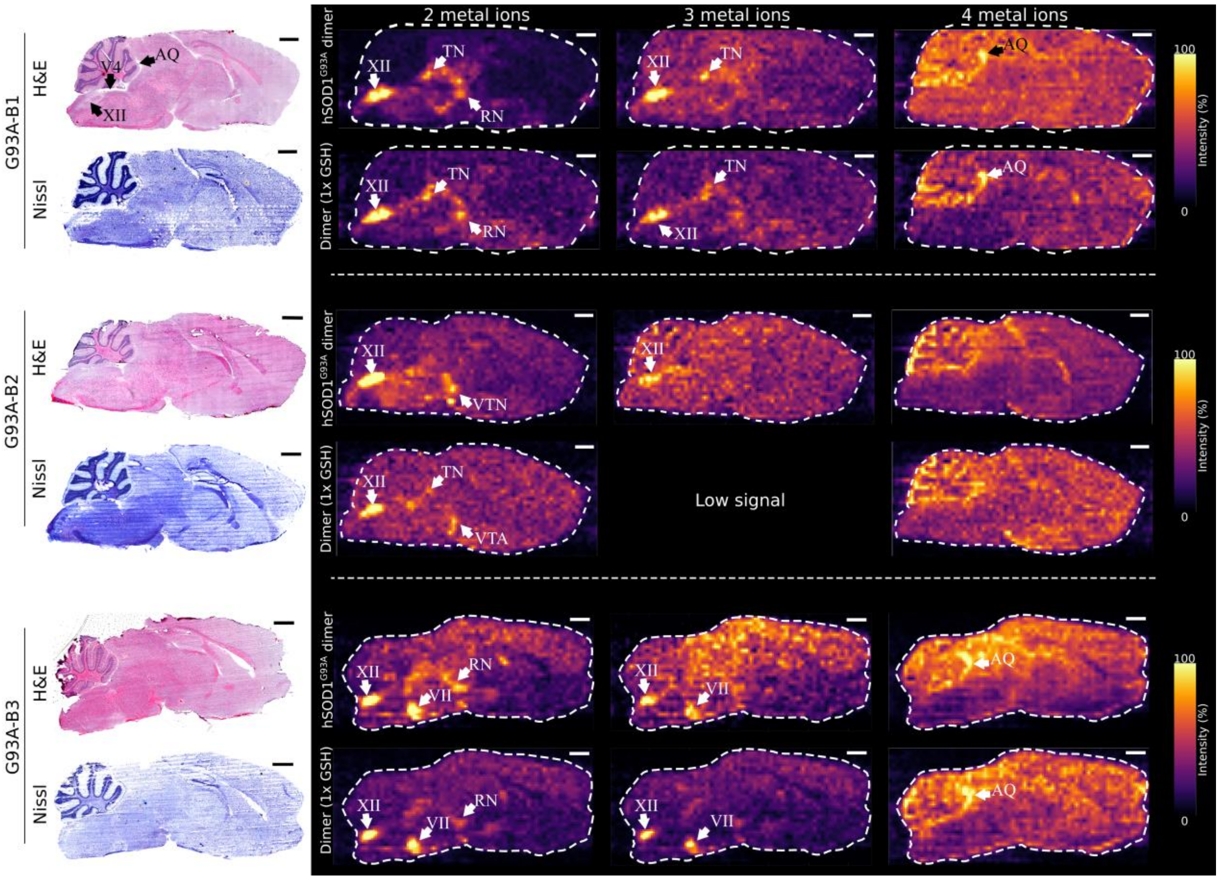

Figure S24: H&E stain, Nissl stains and ion images of hSOD1^{G93A} dimers in G93A-B1, B2 and B3 tissue sections. Ion images are composed of signals from 11+ - 9+ charge states. XII; hypoglossal nucleus, TN; tegmental nuclei, RN; red nucleus, VTA; ventral tegmental area, VII; facial nucleus**.** Scale bar: 1 mm.

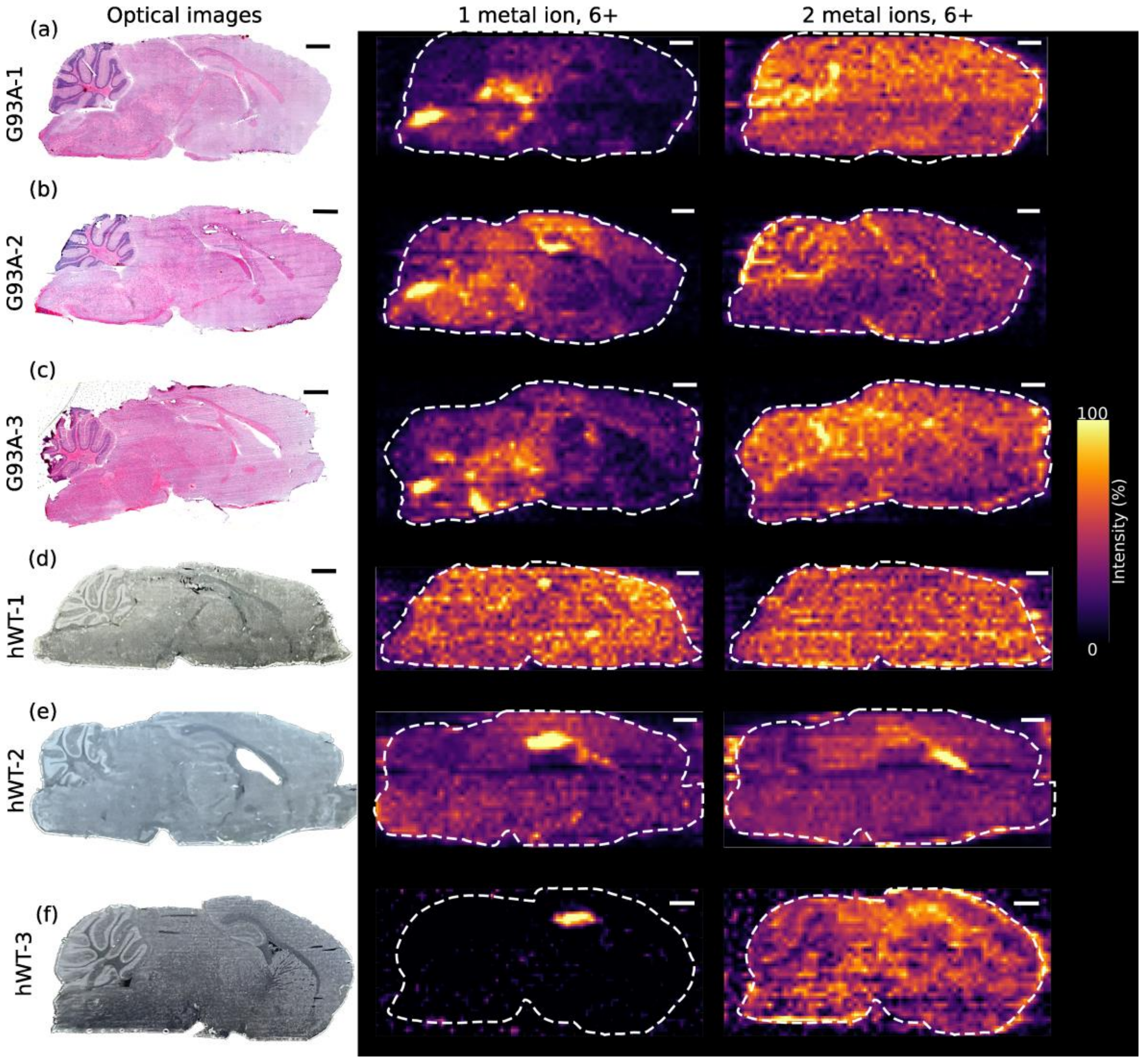

Figure S25: Optical images and ion images for the monomeric 6+ charge state of hSOD1 in (a) G93A-1 (b) G93A-2, (c) G93A-3, (d) hWT-1, (e) hWT-2 and (f) hWT-3.

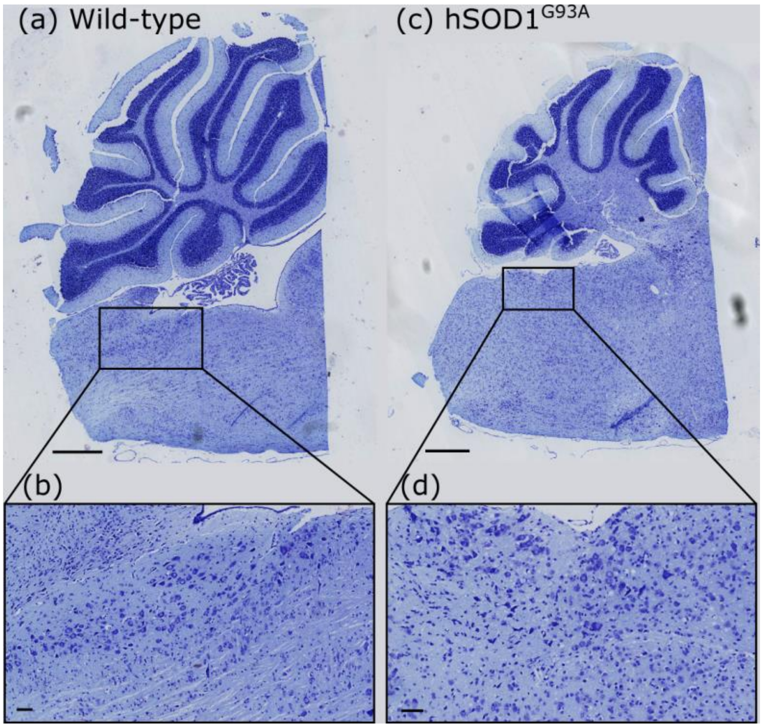

Figure S26: representative FFPE brain sections from mSOD1^{wt} (representative of 12 sections) and hSOD1^{G93A} (representative of 15 sections) mice at 120 days of age. Scale bar (a, c) = 500 μm, (b, d) 50 μm.

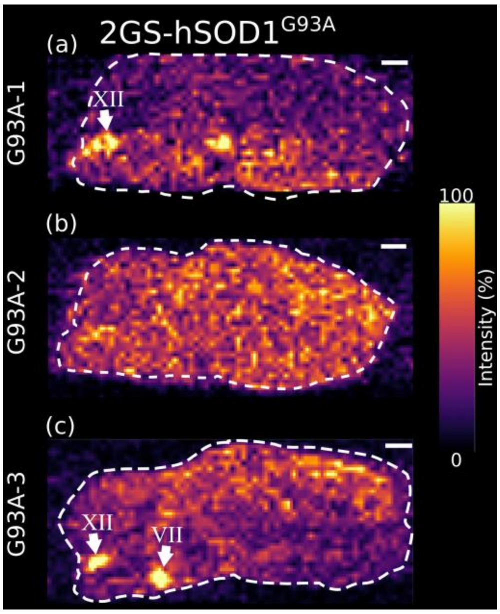

Figure S27: ion images for dimers containing two GS-hSOD1^{G93A} subunits in the 10+ charge state in G93A brains. (a) G93A-1, (b) G93A-2 and (c) G93A-3. A distribution within motor-associated regions was not detected for B2 because of low signal intensity. XII; hypoglossal nucleus, VII; facial nucleus**.** Scale bar 1 mm.

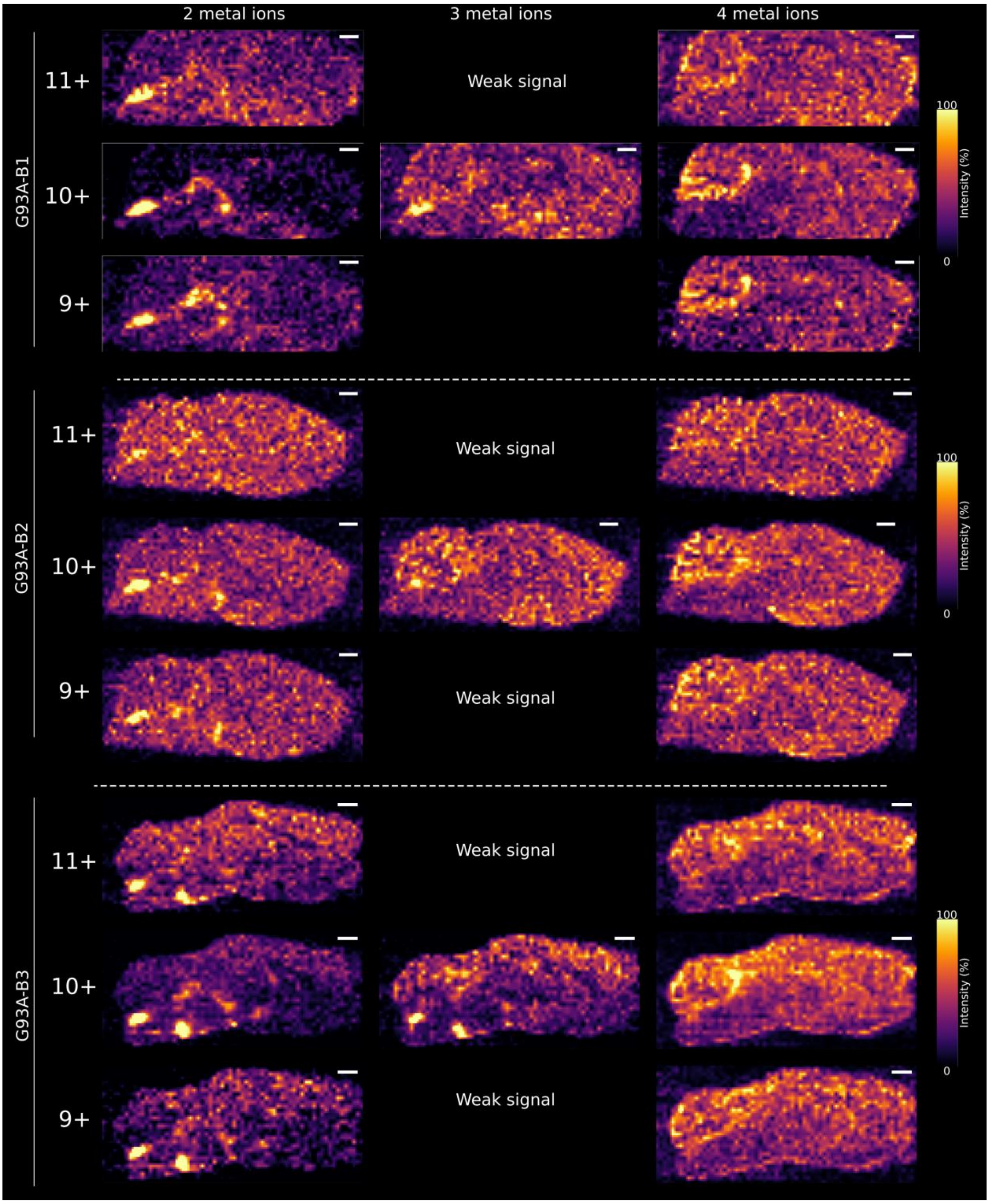

Figure S28: Ion images for 1GS-hSOD1^{G93A} dimers in charge states 11+ - 9+, which include one glutathionylated subunit, for the three G93A mouse brains. Scale bar: 1 mm

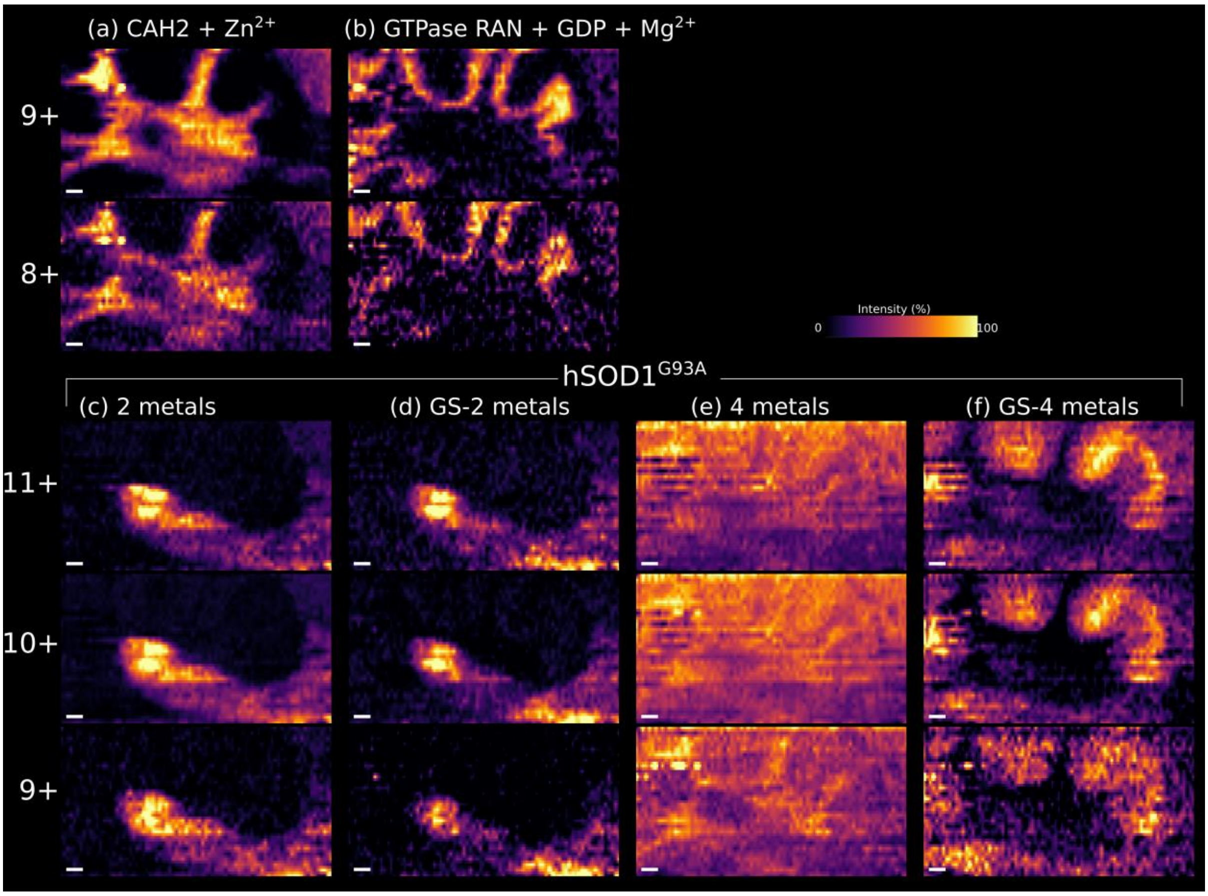

Figure S29: ion images for each charge state of the proteins in the high-resolution MSI of the cerebellum. (a) CAH2 +Zn^{2+} complex and (b) GTPase Ran + GDP + Mg^{2+} complex. hSOD1^{G93A} dimers with (c) 2 metal ions, (d) 2 metal ions and one glutathionylated subunitr, (e) 4 metal ions and (f) 4 metal ions and one glutathionylated subunit. Scale bar: 200 µm.

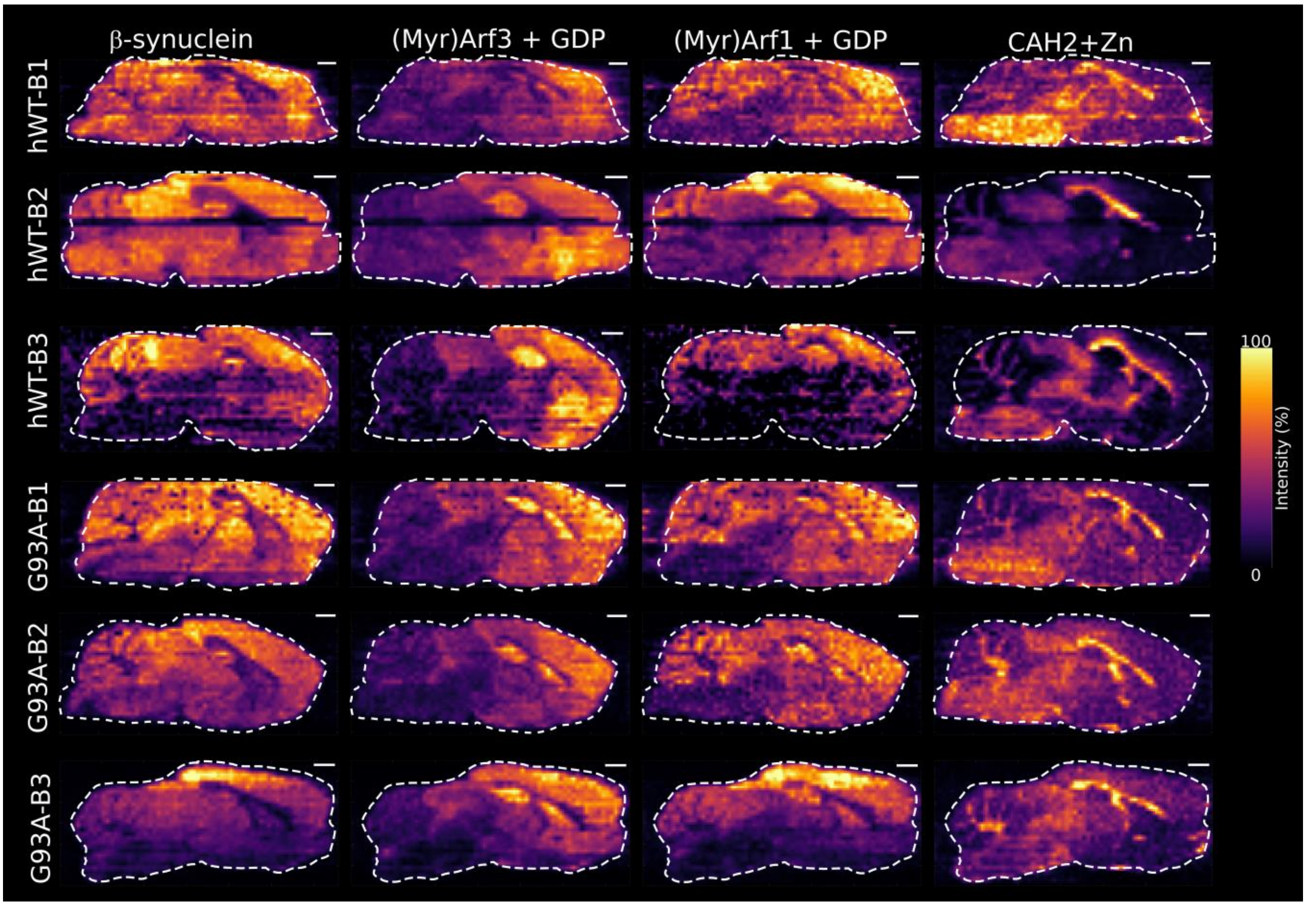

Figure S30: protein for non-SOD1 proteins verify similar performance across experiments. B-synuclein, myristoylated Arf3 bound to guanidine disphosphate (GDP), Myristoylated Arf1 + GDP and carbonic anhydrase 2 bound to Zn^{2+}.

Table S7: Parameters for deconvolution with UniDec.

UniDec parameter	Value
**m/z range**	2545.87 – 3862.62 (full)
**Background subtraction**	Yes
**Charge range**	1-12
**Mass range**	20,000-35,000
**Sample Mass Every (Da)**	1.0
**Peak detection range (Da)**	10.0
**Peak detection threshold**	0.06

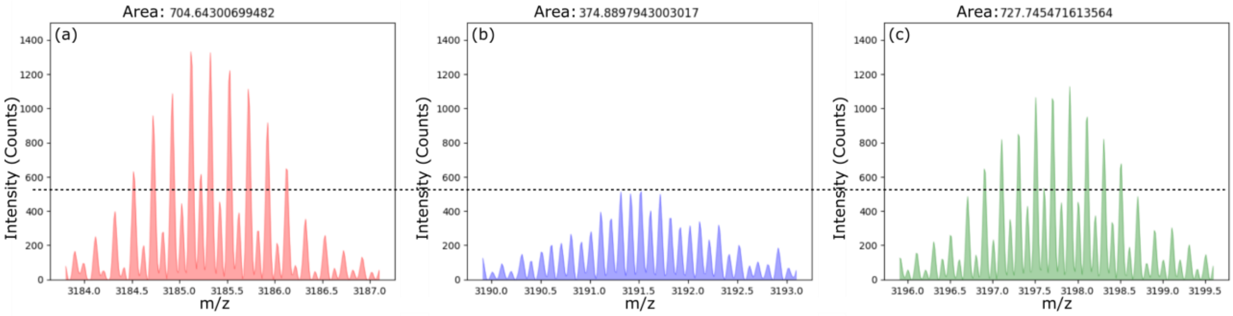

Figure S31: nano-DESI high-resolution MS (orbitrap resolution = 240,000 FWHM at m/z 200) of hSOD1^{G93A} dimer/monomer overlap (10+/5+ charge states). (b) The three-metal dimer has no overlap; the area of this signal was used to estimate the contribution of 10+ dimers ((a) 2 metals and (c) 4 metals) with respect to the total area of their peaks, approx. 52%.

Data availability

The mass spectrometry and optical imaging data generated in this study have been deposited in the University of Birmingham Institutional Research Archive under accession code [10.25500/edata.bham.00001123]. Processed data are also available in the same archive. Mass spectrometry data have also been deposited to the ProteomeXchange Consortium via the PRIDE partner repository with the dataset identifier PXD053247. The signal intensity and motor neuron count data generated in this study are provided in the Source Data file.

References

(1) Roach, P. J.; Laskin, J.; Laskin, A. Nanospray desorption electrospray ionization: an ambient method for liquid-extraction surface sampling in mass spectrometry *Analyst* **2010**, *135*, 2233-2236, 10.1039/c0an00312c

(2) Hale, O. J.; Cooper, H. J. Native Mass Spectrometry Imaging of Proteins and Protein Complexes by Nano-DESI *Anal Chem* **2021**, *93*, 4619-4627, 10.1021/acs.analchem.0c05277

(3) Kertesz, V.; Van Berkel, G. J. Fully automated liquid extraction-based surface sampling and ionization using a chip-based robotic nanoelectrospray platform *J Mass Spectrom* **2010**, *45*, 252-260, 10.1002/jms.1709

(4) Sarsby, J.; Martin, N. J.; Lalor, P. F.; Bunch, J.; Cooper, H. J. Top-down and bottom-up identification of proteins by liquid extraction surface analysis mass spectrometry of healthy and diseased human liver tissue *J Am Soc Mass Spectrom* **2014**, *25*, 1953-1961, 10.1007/s13361-014-0967-z

(5) Chen, J.; Shiyanov, P.; Zhang, L.; Schlager, J. J.; Green-Church, K. B. Top-down characterization of a native highly intralinked protein: concurrent cleavages of disulfide and protein backbone bonds *Anal Chem* **2010**, *82*, 6079-6089, 10.1021/ac1006766

(6) Marty, M. T.; Baldwin, A. J.; Marklund, E. G.; Hochberg, G. K.; Benesch, J. L.; Robinson, C. V. Bayesian deconvolution of mass and ion mobility spectra: from binary interactions to polydisperse ensembles *Anal Chem* **2015**, *87*, 4370-4376, 10.1021/acs.analchem.5b00140

(7) Boyd, S. D.; Ullrich, M. S.; Skopp, A.; Winkler, D. D. Copper Sources for Sod1 Activation *Antioxidants (Basel)* **2020**, *9*, 10.3390/antiox9060500
