# Supplementary material for: Mass spectrometry imaging of SOD1 protein-metal complexes in SOD1G93A transgenic mice implicates demetalation with pathology
Source: Nat Commun. 2024 Aug 8;15:6518. doi: 10.1038/s41467-024-50514-7 (PMC11310518; doi:10.1038/s41467-024-50514-7)
Supplement: Supplementary file 2 — Reporting Summary [file 41467_2024_50514_MOESM2_ESM.pdf]

nature portfolio

Corresponding author(s): HJ Cooper, RJ Mead

Last updated by author(s): Jul 1, 2024

Reporting Summary

Nature Portfolio wishes to improve the reproducibility of the work that we publish. This form provides structure for consistency and transparency in reporting. For further information on Nature Portfolio policies, see our Editorial Policies and the Editorial Policy Checklist.

Statistics

For all statistical analyses, confirm that the following items are present in the figure legend, table legend, main text, or Methods section.

n/a	Confirmed	
☐	☒	The exact sample size (*n*) for each experimental group/condition, given as a discrete number and unit of measurement
☐	☒	A statement on whether measurements were taken from distinct samples or whether the same sample was measured repeatedly
☐	☒	The statistical test(s) used AND whether they are one- or two-sided<br>*Only common tests should be described solely by name; describe more complex techniques in the Methods section.*
☒	☐	A description of all covariates tested
☒	☐	A description of any assumptions or corrections, such as tests of normality and adjustment for multiple comparisons
☐	☒	A full description of the statistical parameters including central tendency (e.g. means) or other basic estimates (e.g. regression coefficient) AND variation (e.g. standard deviation) or associated estimates of uncertainty (e.g. confidence intervals)
☐	☒	For null hypothesis testing, the test statistic (e.g. *F*, *t*, *r*) with confidence intervals, effect sizes, degrees of freedom and *P* value noted<br>*Give P values as exact values whenever suitable.*
☒	☐	For Bayesian analysis, information on the choice of priors and Markov chain Monte Carlo settings
☒	☐	For hierarchical and complex designs, identification of the appropriate level for tests and full reporting of outcomes
☒	☐	Estimates of effect sizes (e.g. Cohen's *d*, Pearson's *r*), indicating how they were calculated

Our web collection on statistics for biologists contains articles on many of the points above.

Software and code

Policy information about availability of computer code

Data collection	Orbitrap Eclipse Tune v4.0 (Thermo Fisher Scientific)
Data analysis	FreeStyle v1.8 SP2 (Thermo Fisher Scientific), MSiReader v1.02 (North Carolina State University, NC), FireFly v3.2.0.23 (Prosolia, Inc), MATlab script "Sum_matlab_figures" (https://doi.org/10.5281/zenodo.12572632, University of Birmingham), UniDec v6.0.4, Microsoft Excel 2024.

For manuscripts utilizing custom algorithms or software that are central to the research but not yet described in published literature, software must be made available to editors and reviewers. We strongly encourage code deposition in a community repository (e.g. GitHub). See the Nature Portfolio guidelines for submitting code & software for further information.

Data

Policy information about availability of data

All manuscripts must include a data availability statement. This statement should provide the following information, where applicable:

- Accession codes, unique identifiers, or web links for publicly available datasets
- A description of any restrictions on data availability
- For clinical datasets or third party data, please ensure that the statement adheres to our policy

The mass spectrometry and optical imaging data generated in this study have been deposited in the University of Birmingham Institutional Research Archive under accession code [10.25500/edata.bham.00001123]. Processed data are also available in the same archive. Mass spectrometry data have also been deposited to the ProteomeXchange Consortium via the PRIDE partner repository with the dataset identifier PXD053247.

The signal intensity and motor neuron count data generated in this study are provided in the Source Data file.

Research involving human participants, their data, or biological material

Policy information about studies with human participants or human data. See also policy information about sex, gender (identity/presentation), and sexual orientation and race, ethnicity and racism.

Reporting on sex and gender	n/a
Reporting on race, ethnicity, or other socially relevant groupings	n/a
Population characteristics	n/a
Recruitment	n/a
Ethics oversight	n/a

Note that full information on the approval of the study protocol must also be provided in the manuscript.

Field-specific reporting

Please select the one below that is the best fit for your research. If you are not sure, read the appropriate sections before making your selection.

☒ Life sciences ☐ Behavioural & social sciences ☐ Ecological, evolutionary & environmental sciences

For a reference copy of the document with all sections, see nature.com/documents/nr-reporting-summary-flat.pdf

Life sciences study design

All studies must disclose on these points even when the disclosure is negative.

Sample size	No calculation performed. Mass Spectrometry Imaging: Sample size selected based on previous experience. Standard resolution: Three biological replicates for brain sections. Two biological replicates for spinal cord sections. High resolution: One biological replicate for brain section and one biological replicate for spinal cord. Each pixel comprises multiple mass spectra. Biological replicates demonstrate trends across individuals. Neuron counting: Five biological replicates are sufficient to demonstrate motor neuron degeneration or lack thereof.
Data exclusions	No data excluded.
Replication	All attempts at replication were successful.
Randomization	Randomisation was not applicable as sample sizes were not large enough.
Blinding	No blinding was performed. Blinding was not possible because signals within the mass spectra effectively unblind the researcher. MS imaging samples were processed and analyzed in a randomised order to prevent bias due to instrument performance.

Reporting for specific materials, systems and methods

We require information from authors about some types of materials, experimental systems and methods used in many studies. Here, indicate whether each material, system or method listed is relevant to your study. If you are not sure if a list item applies to your research, read the appropriate section before selecting a response.

Materials & experimental systems

n/a	Involved in the study	
☒	☐	Antibodies
☒	☐	Eukaryotic cell lines
☒	☐	Palaeontology and archaeology
☐	☒	Animals and other organisms
☒	☐	Clinical data
☒	☐	Dual use research of concern
☒	☐	Plants

Methods

n/a	Involved in the study	
☒	☐	ChIP-seq
☒	☐	Flow cytometry
☒	☐	MRI-based neuroimaging

Animals and other research organisms

Policy information about studies involving animals; ARRIVE guidelines recommended for reporting animal research, and Sex and Gender in Research

Laboratory animals	Mice; SOD1G93A C57BL/6 (120 -130 days); 02298 (120-180 days); wt (120 days).<br>SOD1G93A C57BL/6 transgenic mice, originally B6SJL-Tg (SOD1-G93A)1Gur/J (stock number 002726) mice were obtained from Jackson Laboratory and backcrossed onto the C57BL/6 OlaHsd background (Harlan UK, C57BL/6 J OlaHsd) for at least 20 generations to create the SOD1G93A C57BL/6 transgenic line on an inbred genetic background.<br>Human SOD1wt mice (B6.Cg-Tg(SOD1)2Gur/J) were obtained from the Jackson Laboratory, strain number 0229820, and these were backcrossed onto the C57BL/6 OlaHsd background (Harlan UK, C57BL/6 J OlaHsd) for at least 20 generations.
Wild animals	n/a
Reporting on sex	Sex was not considered in study design. Sex is reported in Table S1.<br>SOD1 G93A mice: 1M, 9F<br>02298: 3M<br>wt: 5F
Field-collected samples	Study did not involve samples collected from the field.
Ethics oversight	All studies using animals were carried out in accordance with the UK Animals (Scientific Procedures) Act 1986 and all procedures were carried out under a Home Office project licence, reviewed and approved by the local ethics committee (University of Sheffield Animal Welfare and Ethical Review Body).

Note that full information on the approval of the study protocol must also be provided in the manuscript.

Plants

Seed stocks	n/a
Novel plant genotypes	n/a
Authentication	n/a
